# Neuronal Population Activity in Macaque Visual Cortices Dynamically Changes through Repeated Fixations in Active Free Viewing

**DOI:** 10.1523/ENEURO.0086-23.2023

**Published:** 2023-10-18

**Authors:** Yukako Yamane, Junji Ito, Cristian Joana, Ichiro Fujita, Hiroshi Tamura, Pedro E. Maldonado, Kenji Doya, Sonja Grün

**Affiliations:** 1 Graduate School of Frontier Biosciences, Osaka University, Osaka 565-0871, Japan; 2 Okinawa Institute of Science and Technology Graduate University, Okinawa 904-0495, Japan; 3 Institute of Neuroscience and Medicine (INM-6 and INM-10) and Institute for Advanced Simulation (IAS-6), Jülich Research Centre, Jülich 52425, Germany; 4 CAS Key Laboratory of Theoretical Physics, Institute of Theoretical Physics, Chinese Academy of Sciences, Beijing 100190, China; 5 Center for Information and Neural Networks (CiNet), National Institute of Information and Communications Technology, Osaka University, Osaka 565-0871, Japan; 6 Department of Neuroscience and Instituto de Neurosciencia Biomedica (BNI), Facultad de Medicina, Universidad de Chile, Santiago 8380453, Chile; 7 Theoretical Systems Neurobiology, Rheinisch Westfaelische Technische Hochschule (RWTH) Aachen University, Aachen 52056, Germany

**Keywords:** object recognition

## Abstract

During free viewing, we move our eyes and fixate on objects to recognize the visual scene of our surroundings. To investigate the neural representation of objects in this process, we studied individual and population neuronal activity in three different visual regions of the brains of macaque monkeys (*Macaca fuscata*): the primary and secondary visual cortices (V1, V2) and the inferotemporal cortex (IT). We designed a task where the animal freely selected objects in a stimulus image to fixate on while we examined the relationship between spiking activity, the order of fixations, and the fixated objects. We found that activity changed across repeated fixations on the same object in all three recorded areas, with observed reductions in firing rates. Furthermore, the responses of individual neurons became sparser and more selective with individual objects. The population activity for individual objects also became distinct. These results suggest that visual neurons respond dynamically to repeated input stimuli through a smaller number of spikes, thereby allowing for discrimination between individual objects with smaller energy.

## Significance Statement

Conventionally, ventral visual neurons, which are crucial for object recognition, have been studied under passive viewing conditions that involve forced fixation in fixation tasks. However, the importance of investigating visual perception under active conditions is gaining recognition. We studied individual and population neuronal activity in three different ventral visual areas of the brains of macaque monkeys, primary and secondary visual cortices (V1, V2) and inferotemporal cortex (IT), during free viewing, focusing on the relationship between spiking activity, the order of fixations, and the fixated objects. We found that the population activity for individual objects became distinct across repeated fixations. These results suggest that neurons change their population activity depending on sensory experiences and thus raises a question about how visual sensory neurons shape their selectivity.

## Introduction

To visually recognize our surrounding environments, we repeat fixations and saccades. This strategy of our visual system is thought to stem from our retinal structure, which has substantially more cone receptors in the fovea than in the periphery. By moving our eyes to project an image of a target object onto the fovea, we can see the object in high resolution (Land and Tatler, 2009). This unique strategy requires our visual processing and eye movement systems to cooperate in a specific manner. Therefore, studying sensory and motor processing in a unified framework (O’Regan and Noë, 2001; Schroeder et al., 2010; Gegenfurtner, 2016) is crucial for understanding vision under natural conditions. Various studies on the relationship between eye movement and vision (for review, see Kowler, 2011) have indicated that sensory processing is never independent of motor action. However, such studies have mainly focused on dorsal pathway neurons and examined neural activity using simple stimuli, such as dots or Gabor patches, under task conditions where subjects are forced to make saccades in a particular way. In contrast, physiological studies of object recognition and representation in ventral pathway neurons are conventionally performed under forced fixation conditions. The implicit assumption has been that passive visual stimuli (mostly in a random order) are processed in the same way as actively explored visual stimuli (but see Sheinberg and Logothetis, 2001; Ito et al., 2022). However, growing evidence and theoretical considerations show the importance of investigations into visual processing under active vision conditions (Leopold and Park, 2020; Huber-Huber et al., 2021).

Moreover, under active vision conditions, an observer sequentially samples the input stimuli through eye movements. Sampling history (i.e., what has been seen previously) is an important factor in shaping visual neural activity patterns. It has been shown that short-term modulations of neuronal activity occur across the ventral visual pathway in an experience-dependent manner. A prominent modulation is adaptation, in which repeated presentation of the same stimulus reduces the neural response (Miller et al., 1991; Ringo, 1996; Peter et al., 2021). There are multiple views on how this phenomenon should be interpreted (Desimone, 1996; McMahon and Olson, 2007; De Baene and Vogels, 2010; Gotts et al., 2012; Brunet et al., 2014; Auksztulewicz and Friston, 2016; Grotheer and Kovács, 2016). Furthermore, the impact of such experience-dependent modulations of neuronal activity on population coding is still unclear. Therefore, to understand visual object recognition, it is important to examine whether such modulations happen in realistic active viewing conditions and to identify their effects on population activity.

In this study, we explored population neuronal activity in macaque monkeys during active vision. The experiments were conducted under natural sampling conditions, with the animals free to decide on the objects in the stimulus image to fixate on (free viewing). We recorded the neuronal activities of the primary (V1) and secondary (V2) visual cortices (lower visual areas) and the inferotemporal (IT) cortex, and examined changes in neuronal activity with fixation order. We found that the responses of individual neurons became sparser in later fixations and that the population representation of individual objects became more distinct across repeated fixations.

## Materials and Methods

### Animal preparation

We used two female macaque monkeys (*Macaca fuscata*; body weights, 7.1 and 5.2 kg), which were maintained in individual housings. A head restraint and two recording chambers for electrophysiological recordings, one for V1/V2 and the other for IT, were implanted in the skull of each monkey. The recording position in V1/V2 was adjusted so that the receptive fields of the neurons were close to the fovea and object images fell in the receptive fields when they were fixated on. The recording position in IT ranged from 0.5 to 9.0 mm for monkey 1 and 2.6 to 11 mm for monkey 2, anterior from the auditory meatus, and included the lateral convexity of IT and the superior temporal sulcus. The surgical method has been described elsewhere (Ito et al., 2017, 2022). Briefly, the surgery was performed under full anesthesia by inhalation of 1–3% isoflurane (Forane; Abbott Japan) in nitrous oxide (70% N_2_O, 30% O_2_) through an intratracheal cannula. An antibiotic (40 mg; Piperacillin Sodium Toyama Chemical; administered by intramuscular injection), and anti-inflammatory and analgesic agents (12.5 mg of Voltaren; Novartis; 0.6 mg/kg ketoprofen; Nissin Pharmaceutical; both administered by intramuscular injection), were given immediately after the surgery and continued during the first postoperative week. After one to two weeks of recovery, a scleral search coil, used for measuring eye positions, was implanted in the left eye under the same anesthesia mentioned above. After recovery from surgery, we trained the animals for the tasks for approximately two weeks. Two linear array electrodes were inserted into V1/V2 and IT for recordings. All the experiments were performed according to the guidelines from National Institutes of Health (1996) and the Japan Neuroscience Society, and were approved by the animal experiment committee of Osaka University (certification No. FBS-13-003).

### Behavioral tasks

During the testing, a monkey sat in a chair with its head fixed. A liquid-crystal display monitor (FlexScan EV2736W-FS; EIZO) was placed 570 mm away from the monkey’s eyes for stimulus presentation. The monkeys were trained to perform eye calibration, fixation, and free viewing tasks, as described later. A drop of water was delivered after a successful trial. We defined a “session” as a set of trials for a particular task, such as free viewing, with a particular stimulus set. The typical sequence for a recording session in a day was as follows: (1) an eye calibration task session, (2) several fixation task sessions to determine the recording depth of the electrodes, (3) a fixation task session to examine object selectivity in IT, (4) an eye calibration task session, (5) free viewing task sessions, (6) an eye calibration task session, and (7) a fixation task session to examine the orientation selectivity of neurons in V1 and V2.

#### Eye calibration task

The eye calibration task was performed at the beginning of each recording day. In most cases, this task was also performed before and after the main free viewing task sessions to ensure precise estimation of the eye position during free viewing. In the eye calibration task, a square fixation point (0.2°) was shown at one of nine positions on a three-by-three square grid extending 38° × 28°, which was larger than the stimulus image for the free viewing task. The monkey was required to fixate within 0.5° of the radius around each fixation point for 800 ms to complete a trial. After each trial, the monkey received drops of water or juice as a reward. The task continued until three successful fixation trials were completed for each position. The average vertical and horizontal voltage values from the search coil system during fixation were used to calculate the transformation functions for determining the gaze positions.

#### Fixation task for the determination of recording depth and object selectivity

Conventional fixation tasks were performed to determine the recording depth and quickly examine the stimulus selectivity of IT neurons. Current source density (CSD) analysis of the visually evoked local field potentials (LFPs) was performed to determine the depth of the signal-pickup probes along a linear array electrode (Self et al., 2013). Additionally, based on the CSD signal, we identified the positions of the electrodes in the white matter separating V1 and V2. A trial started with the presentation of a fixation spot (0.2° square) at the center of the monitor screen, followed by sequential presentations of object images, each for 200 ms, with a 200-ms interstimulus interval. After each trial, the monkey received drops of water or juice as a reward. We used the same 64 object stimuli used for the free viewing task (see below, Visual stimuli for free viewing). Typically, six stimuli were presented sequentially in a trial. For the fixation task shown in Extended Data Figure 5-1, we used the same 20 object images that were used for the free viewing task sessions on the same recording day. Twenty stimuli were shown 10 times, yielding 200 stimuli. Because the order of the stimuli was randomized in each repeat, most consecutive stimuli differed.

#### Free viewing task

**Figure 1.**
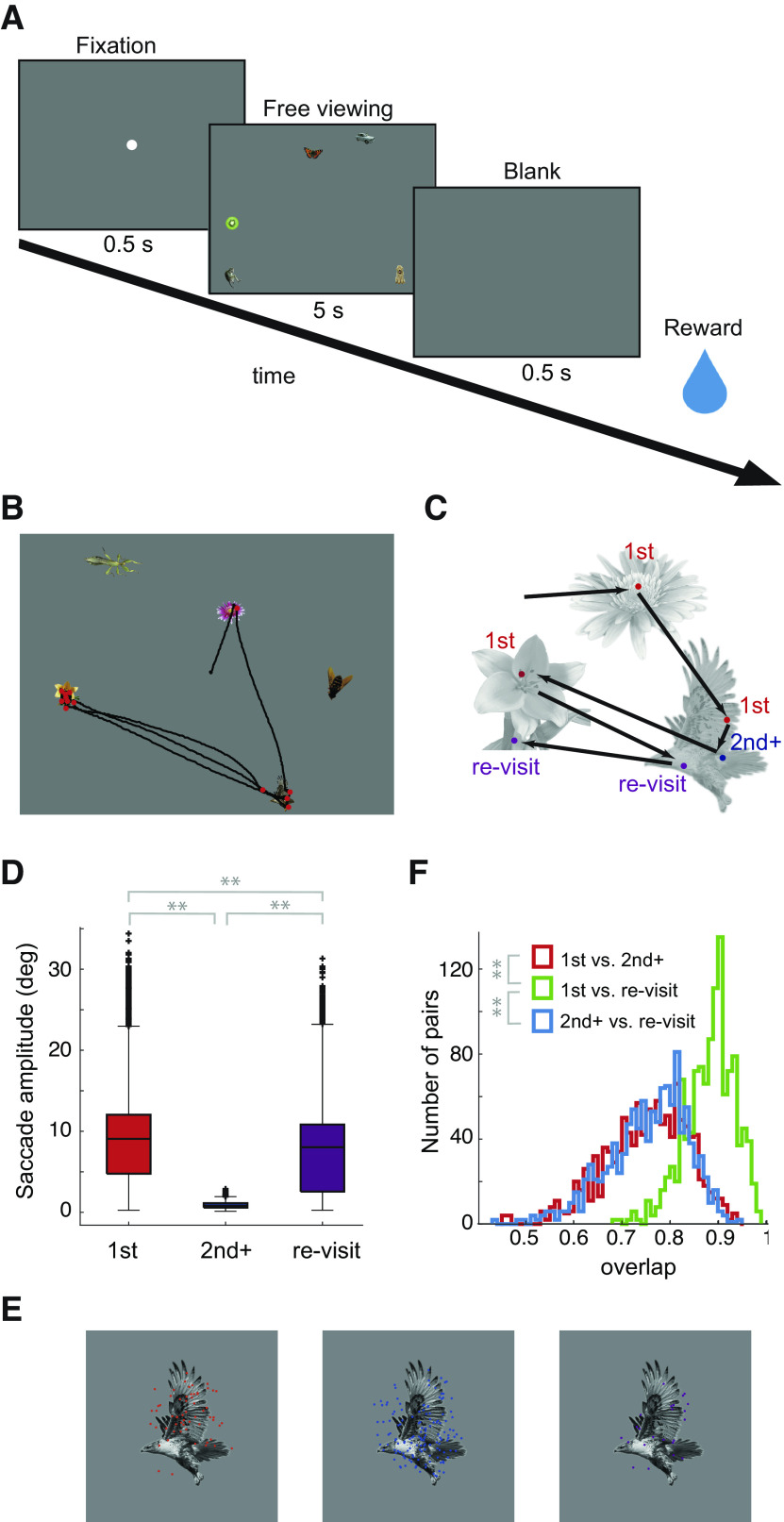
***A***, Free viewing task paradigm. The trial starts with the appearance of a fixation spot. The animal needs to fixate on the spot for 0.5 s (here, the fixation spot size is exaggerated). After completion of fixation, an image for free viewing appears. The animal is required to keep its eyes within the monitor screen for 5 s. The liquid reward is delivered after completion of the 5-s free viewing. ***B***, Example visual stimulus image and corresponding eye movement pattern (black lines) and fixation positions (red dots) during one stimulus image presentation. ***C***, Categorization of fixations. ***D***, Saccade amplitude just before the first, second+, and re-visit fixations (*n* = 91,474, 65,730, and 45,289 for the first, second+, and re-visit, respectively). Horizontal lines are the medians, the upper and lower borders of the boxes are the 25th-75th percentiles, and the upper and lower whiskers represent the maximum and minimum values of nonoutliers. Crosses are outliers. Asterisks indicate statistical significance for the Mann–Whitney *U* test (*p*-value = 0 for first vs. second+, 0 for first vs. re-visit, and 0 for second+ vs. re-visit). Extended Data Table 1-1 summarizes the related statistical values. ***E***, Example fixation positions of an object in first (red), second+ (blue), and re-visit (purple) fixations. ***F***, Distribution of fixation position overlaps of the same object between different fixation categories (*n* = 1176). Asterisks indicate statistical significance for the sign-rank test (*p*-value = 5.8 × 10^−182^ for first-second+ vs. first-re-visit and 6.0 × 10^−184^ for first-re-visit vs. second+-re-visit). Extended Data Table 1-2 summarizes the related statistical values.

A trial started with the presentation of a fixation spot (0.2° square) at the center of the monitor screen (Fig. 1*A*). Once the animal had fixated on the fixation spot for 0.5 s, the spot disappeared and a stimulus image for free viewing (see below) appeared. Then, the animal was allowed to view the stimulus image freely, but not allowed to direct their gaze outside the boundaries of the image, which led to the abortion of the trial with no reward. The image was turned off if the animal successfully viewed the image for 5.0 s, and a reward was delivered 0.5 s later. The intertrial interval was 2.0 s (for monkey 1) or 1.5 s (for monkey 2). The free viewing task session continued as long as the animal’s motivation lasted (typically >30 min). Usually, two free viewing sessions, one with gray-background stimuli and the other with stimuli comprising a natural background scene, were performed daily. In this report, we only analyzed the sessions that used the gray-background stimuli.

10.1523/ENEURO.0086-23.2023.tab1-1 Extended Data Table 1-1 Comparison of saccade amplitude. The *p*-values were determined by the rank-sum test (two sided). The effect size is the Cliff’s δ effect size. Download Table 1-1, DOCX file.

10.1523/ENEURO.0086-23.2023.tab1-2 Extended Data Table 1-2 Comparison of fixation position overlap. The *p*-values were determined by the signed-rank test (two sided). The effect size is the Cliff’s δ effect size. Download Table 1-2, DOCX file.

### Visual stimuli for free viewing

The stimulus images for the free viewing experiments were generated by placing five object images (hereinafter “object”) on a gray background. On average, the objects were 2° in diameter and taken by one of the authors or selected from the Microsoft image gallery (previously available from Office Online). The total number of objects prepared was 129. Typically, we prepared a set of 64 objects (eight samples from eight different object categories) and used this set as an initial selectivity survey for IT neurons. In each session over the course of a day, 20 objects from the set of 64 were selected based on the multiunit activity (MUA) of IT examined in the fixation task. Five objects were randomly selected out of 20 objects and randomly placed onto a gray background to create a stimulus image. The distance between the objects was larger than 4° so that the objects did not overlap. This was repeated 60 times to generate 60 stimulus images. In a single free viewing task session, each stimulus image was presented three to five times in a pseudo-random sequence (the order of the presented images was randomized). The monkeys became familiar with the objects through the training and multiple recording sessions, despite the placement and combination of the objects being new to the monkeys in each recording session. The objects for the fixation task were the same as the 20 for the free viewing task.

### Recording system and electrodes

The tasks were controlled using a custom-made program on a programmable logic controller (KV5000; KEYENCE). Eye positions were recorded with a scleral search coil system (DSC2000; Sankei Kizai Co, Ltd.). Neuronal activity was recorded using linear array electrodes with 24 channels (V-probe; Plexon Inc.), amplified, and bandpass filtered (0.7–8000 Hz) using a commercial amplifier (Plexon Inc.). Neuronal activity and eye position data were acquired through an analog-digital board (National Instruments) at a sampling rate of 20 kHz, and stored on hard disk drives for offline analysis.

### Data analysis

#### Detection of fixations and saccades

The method of eye event detection is described by Ito et al. (2017). First, we estimated the velocity and acceleration of eye movements by computing the temporal derivatives of the data using the Savitzky-Golay filters (Savitzky and Golay, 1964; Nyström and Holmqvist, 2010). Then, we collected the data from the time segments throughout which the eye velocity and acceleration were above 30 and 8000°/s^2^, respectively, as potential saccade periods. Segments considered to be artifacts or noise were dropped; the criteria for this were a peak velocity exceeding 1500°/s, peak acceleration exceeding 120,000°/s^2^, duration of <5 ms, duration of >100 ms, or gaze shift of <0.1°. We identified the remaining segments as saccade periods, and the start and end times of each segment were stored as saccade onset and offset, respectively. We did not discriminate between microsaccades and regular saccades. Finally, the periods between two successive saccades were identified as fixation periods unless the gaze shift exceeded 1.0°.

#### Behavioral classification of fixations

**Figure 2.**
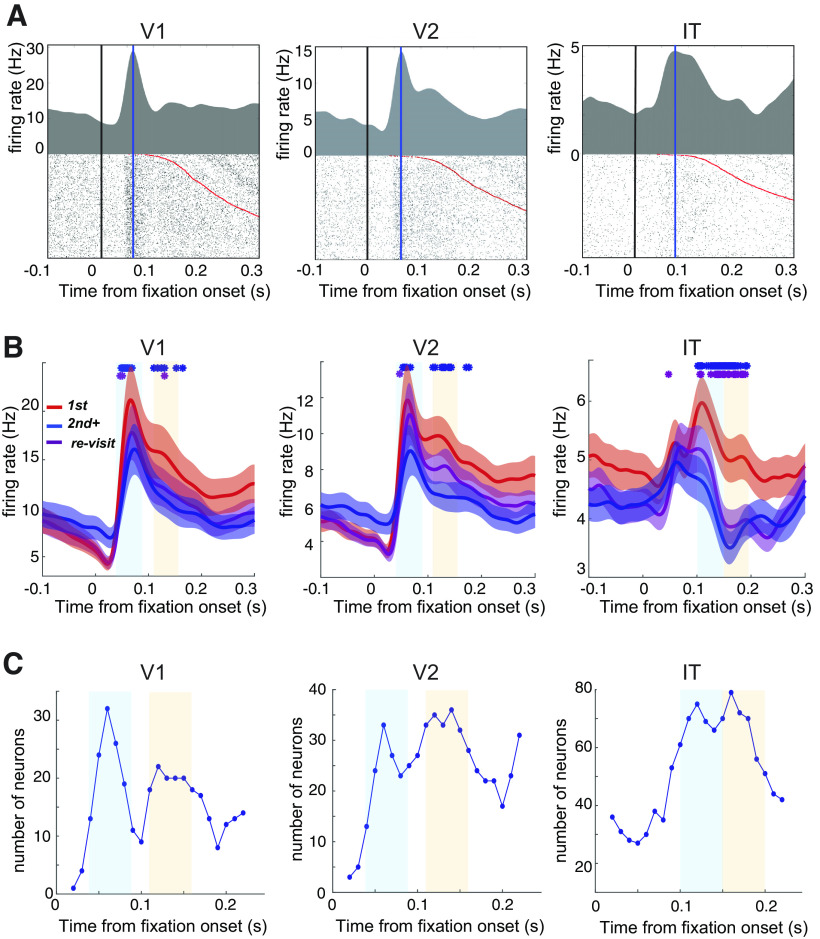
***A***, Fixation-triggered perifixation time histogram (PFTH) and raster plot of example neurons from each area. The vertical blue line is the peak of the PFTH. Red dots in the raster plot are fixation offsets, i.e., the onset of the next saccade. ***B***, Comparison of the firing rates of different fixation categories. The population mean firing rates of recorded neurons in each area. Colored lines (red, purple, and blue) indicate different fixation categories, with the colored shaded areas indicating the SEM (*n* = 87, 92, and 337 for V1, V2, and IT, respectively). Asterisks indicate the time points that showed significant differences in the mean firing rates between first and second+ (blue) or first and re-visit (purple) fixations (bin size 1 ms; signed-rank test, *p *<* *0.01, Bonferroni corrected). For comparison, the response to the preferred object is summarized in Extended Data Figure 2-1. ***C***, Number of neurons that showed significant (*p *<* *0.01, rank-sum test, one-sided) decreases in firing rates between the first and later (second+ and re-visit) fixations across time. The light blue and yellow shaded areas in ***B*** and ***C*** indicate the fixation order-dependent response (FODR) periods 1 and 2, respectively.

**Figure 3.**
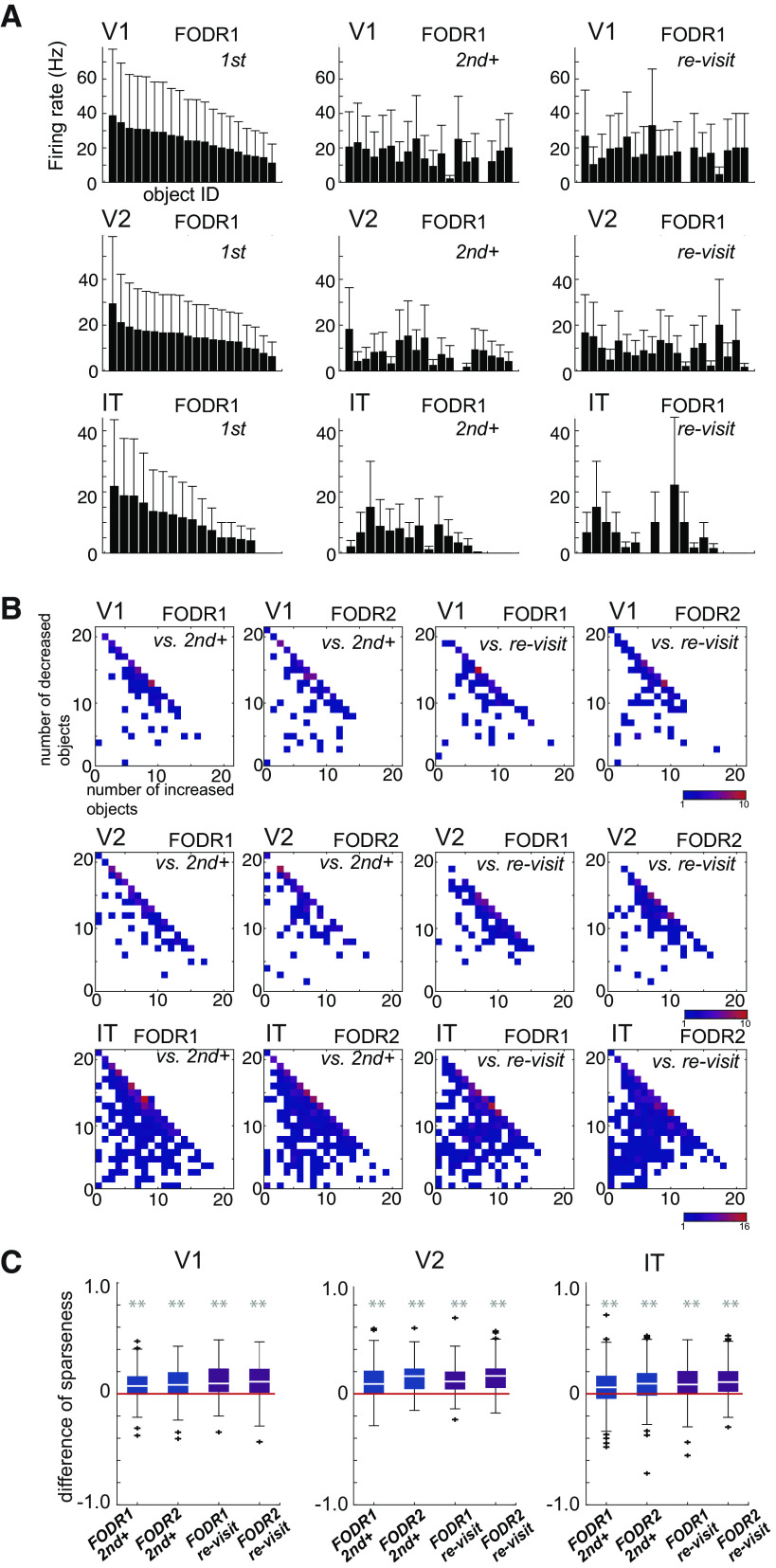
***A***, Object selectivity of example neurons. Each bar is the mean firing rate for fixation on each object during FODR1. The object IDs are arranged in descending order on the *x*-axis according to the mean firing rate for first fixations. The error bars are the SEM. ***B***, The number of objects that evoked increased or decreased responses from first to second+ (left) or from first to re-visit (right) fixations calculated during FODR1 or FODR2 (*n* = 87, 97, and 337 for V1, V2, and IT, respectively). ***C***, Decrease in sparseness in second+ and re-visit fixations compared with first fixations during FODR1 and FODR2. The sparseness of first fixations was subtracted for each neuron. Colors correspond to the fixation orders (*n* = 85, 95, and 318 for V1, V2, and IT, respectively). The convention for the box plots is the same as in Figure 1*D*. Asterisks indicate statistical significance for the signed-rank test between first fixations and second+ or re-visit fixations (**p < 0.01). The *p*-values are 2.4 × 10^−5^, 1.5 × 10^−6^, 1.5 × 10^−4^, and 1.4 × 10^−8^ for FODR1-second+, FODR2-second+, FODR1-re-visit, and FODR2-re-visit, respectively, for V1; 2.1 × 10^−10^, 2.5 × 10^−13^, 6.8 × 10^−13^, and 4.1 × 10^−14^ for FODR1-second+, FODR2-second+, FODR1-re-visit, and FODR2-re-visit, for V2; and 5.5 × 10^−10^, 1.1 × 10^−13^, 5.8 × 10^−24^, and 2.3 × 10^−31^ for IT). Extended Data Tables 3-1 and 3-2 summarize the related statistical values. The comparison of sparseness across recording areas or across subjects is shown in Extended Data Figure 3-1.

**Figure 4.**
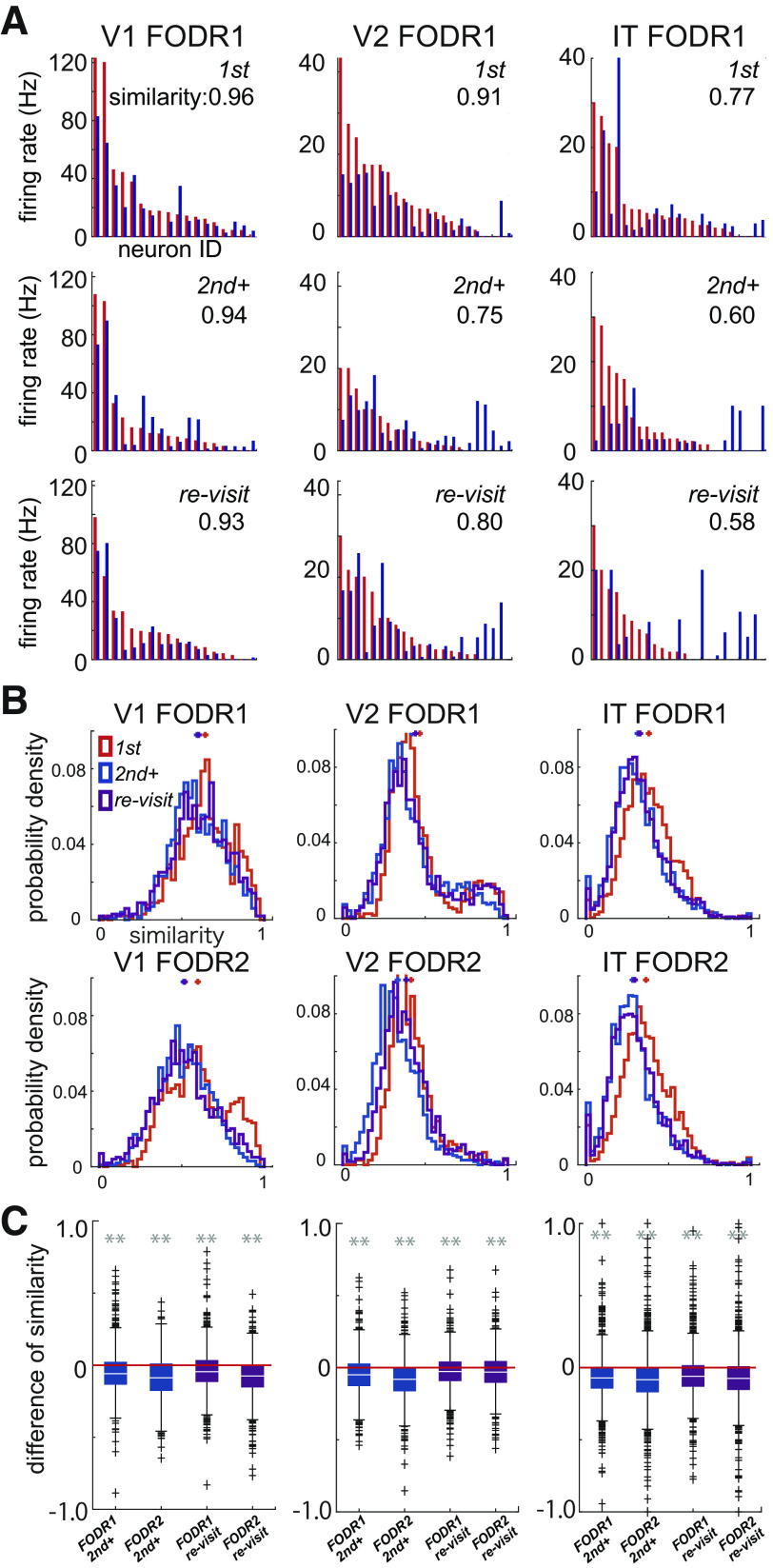
***A***, Population activity profiles of example objects. Red and blue correspond to different objects. Each bar corresponds to the mean firing rate of neurons for an object. The neuronal IDs are arranged in descending order on the *x*-axis according to the mean firing rates for the object shown in the red bar in each fixation category. The cosine similarity of each combination of firing profiles is shown at the top right. ***B***, Cosine similarity distribution calculated for all available object combinations for each fixation category (corresponds to the color; *n* = 991, 1210, and 2342 for V1, V2, and IT, respectively). Crosses at the top are the means of each distribution. ***C***, Decrease in cosine similarity. Cosine similarities for first fixations were subtracted from those of second+ and re-visit fixations. Colors correspond to the fixation orders (*n* = 991, 1210, and 2342 for V1, V2, and IT, respectively). The white lines are the medians and the upper and lower borders of the boxes are the 25th–75th percentiles, and the upper and lower whiskers represent the maximum and minimum values of nonoutliers. Crosses are outliers. Asterisks indicate significant differences for the Kolmogorov–Smirnov test (**p < 0.01). The *p*-values are 1.3 × 10^−14^ (vs. second+ FODR1), 6.4 × 10^−16^ (vs. second+ FODR2), 2.2 × 10^−6^ (vs. re-visit FODR1), and 2.9 × 10^−5^ (vs. re-visit FODR2) for V1; 3.6 × 10^−12^ (vs. second+ FODR1), 1.0 × 10^−54^ (vs. second+ FODR2), 0.8 × 10^−9^ (vs. re-visit FODR1), and 6.3 × 10^−15^ (vs. re-visit FODR2) for V2; and 6.3 × 10^−51^ (vs. second+ FODR1), 2.3 × 10^−78^ (vs. second+ FODR2), 1.2 × 10^−34^ (vs. re-visit FODR1), and 1.1 × 10^−53^ (vs. re-visit FODR2) for IT. Extended Data Tables 4-1 and 4-2 summarize the related statistical values. The comparison of cosine similarity across recording areas or subjects and the effect of reduction of firing rate on cosine similarity is shown in Extended Data Figure 4-1.

**Figure 5.**
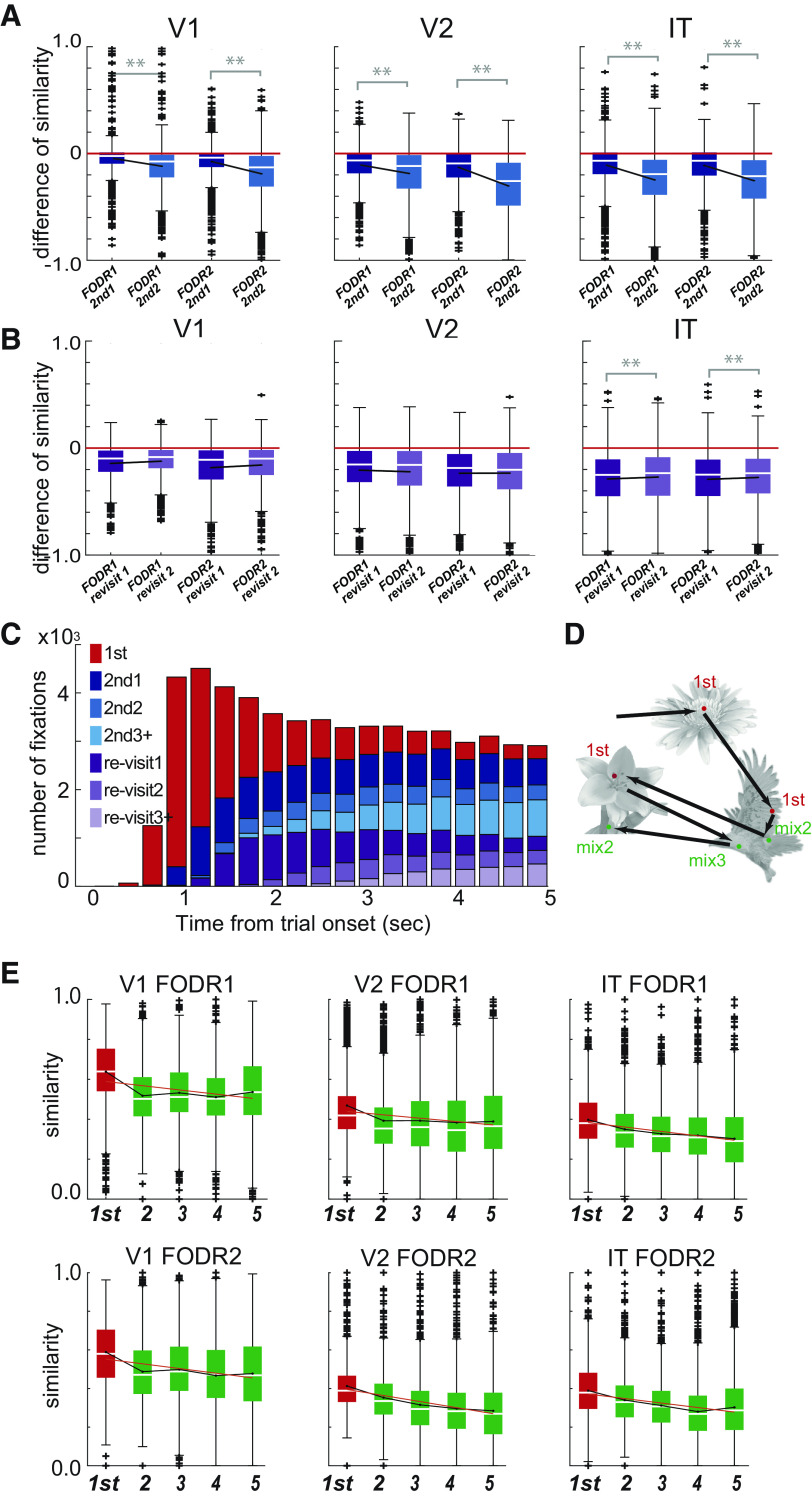
***A***, Comparison of mean cosine similarities between second1 and second2 fixations for the three areas. The box plot shows the cosine similarities for second1 and second2 subtracted from those of the first fixations. Colors correspond to fixation orders. The convention for the box plots is the same as in Figure 1*D*. Asterisks indicate significant differences for the Kolmogorov–Smirnov test (**p < 0.01). The *p*-values are 1.5 × 10^−16^ (second1 vs. second2 in FODR1 of V1), 1.2 × 10^−21^ (FODR2 of V1), 6.9 × 10^−10^ (FODR1 of V2), 1.2 × 10^−39^ (FODR2 of V2), 2.5 × 10^−47^ (FODR1 of IT), and 1.0 × 10^−55^ (FODR2 of IT). The black lines are the means (*n* = 788, 910, and 1832 for V1, V2, and IT, respectively). Extended Data Table 5-1 summarizes the related statistical values. ***B***, Comparison of cosine similarities between re-visit1 and re-visit2 fixations. Conventions are same as in A (*n* = 534, 620, and 1654 for V1, V2, and IT, respectively). The *p*-values are 0.11 (re-visit1 vs. re-visit2 in FODR1 of V1), 0.19 (FODR2 of V1), 0.78 (FODR1 of V2), 1.0 (FODR2 of V2), 0.006 (FODR1 of IT, higher for re-visit2), and 0.01 (FODR2 of IT, higher for re-visit2). Extended Data Table 5-2 summarizes the related statistical values. ***C***, Fixation order distribution within 5.0 s of the onset of the free viewing trial. ***D***, Categorization of fixations by mixed-sample ordering. ***E***, Comparison of cosine similarities among fixation orders in mixed-sample ordering (green). For reference, the cosine similarity for a first fixation is shown in red. The red line is the linear fit to the mean cosine similarity across fixation orders (*n* = 846, 989, and 1835 for V1, V2, and IT, respectively). Extended Data Tables 5-3, 5-4, and 5-5 summarize the related statistical values. For comparison, Extended Data Figure 5-1 summarizes the cosine similarity for the fixation task.

**Figure 6.**
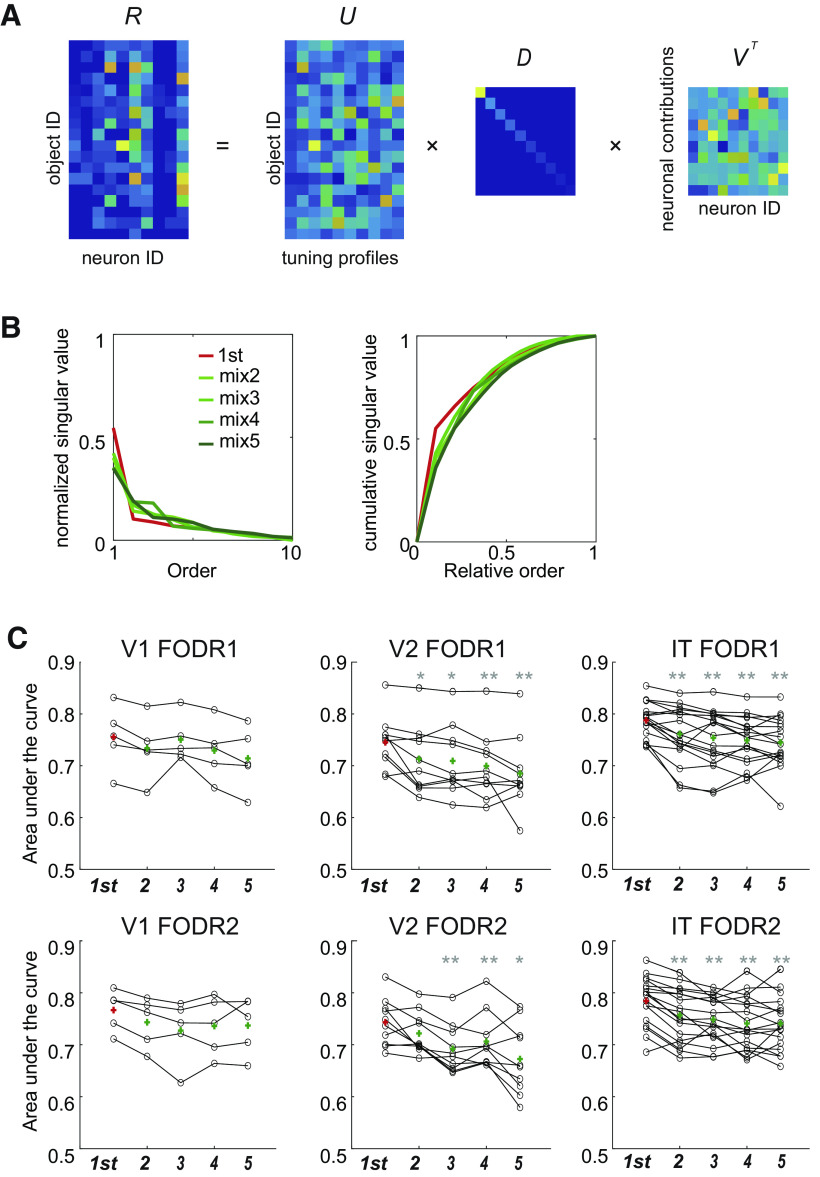
***A***, Singular value decomposition of an example session of IT (data of FODR1, mix2 fixations). ***B***, Normalized singular values (left) and their cumulative values (right) from the same example session as ***A***. Color corresponds to the saccade orders. ***C***, Area under the curve of all the sessions of different fixation categories (first, and mixed 2, 3, 4, and 5). The red and green crosses are the mean AUC across sessions. Asterisks indicate significant differences for the signed-rank test (*p < 0.05; **p < 0.01). The *p*-values are 0.063 (vs. mix2), 0.63 (vs. mix3), 0.063 (vs. mix4), and 0.063 (vs. mix5), for V1 of FODR1; 0.063 (vs. mix2), 0.063 (vs. mix3), 0.063 (vs. mix4), and 0.063 (vs. mix5), for V1 of FODR2; 0.014 (vs. mix2), 0.020 (vs. mix3), 2.0 × 10^−3^ (vs. mix4), and 3.9 × 10^−3^ (vs. mix5) for V2 of FODR1; 0.084 (vs. mix2), 2.0 × 10^−3^ (vs. mix3), 2.0 × 10^−3^ (vs. mix4), and 0.039 (vs. mix5) for V2 of FODR2; 2.1 × 10^−3^ (vs. mix2), 1.6 × 10^−3^ (vs. mix3), 4.6 × 10^−4^ (vs. mix4), and 1.8 × 10^−3^ (vs. mix5) for IT of FODR1; and 5.4 × 10^−4^ (vs. mix2), 1.0 × 10^−3^ (vs. mix3), 2.3 × 10^−4^ (vs. mix4), and 3.3 × 10^−4^ (vs. mix5) for IT of FODR2. Extended Data Tables 6-1 and 6-2 summarize the related statistical values.

**Figure 7.**
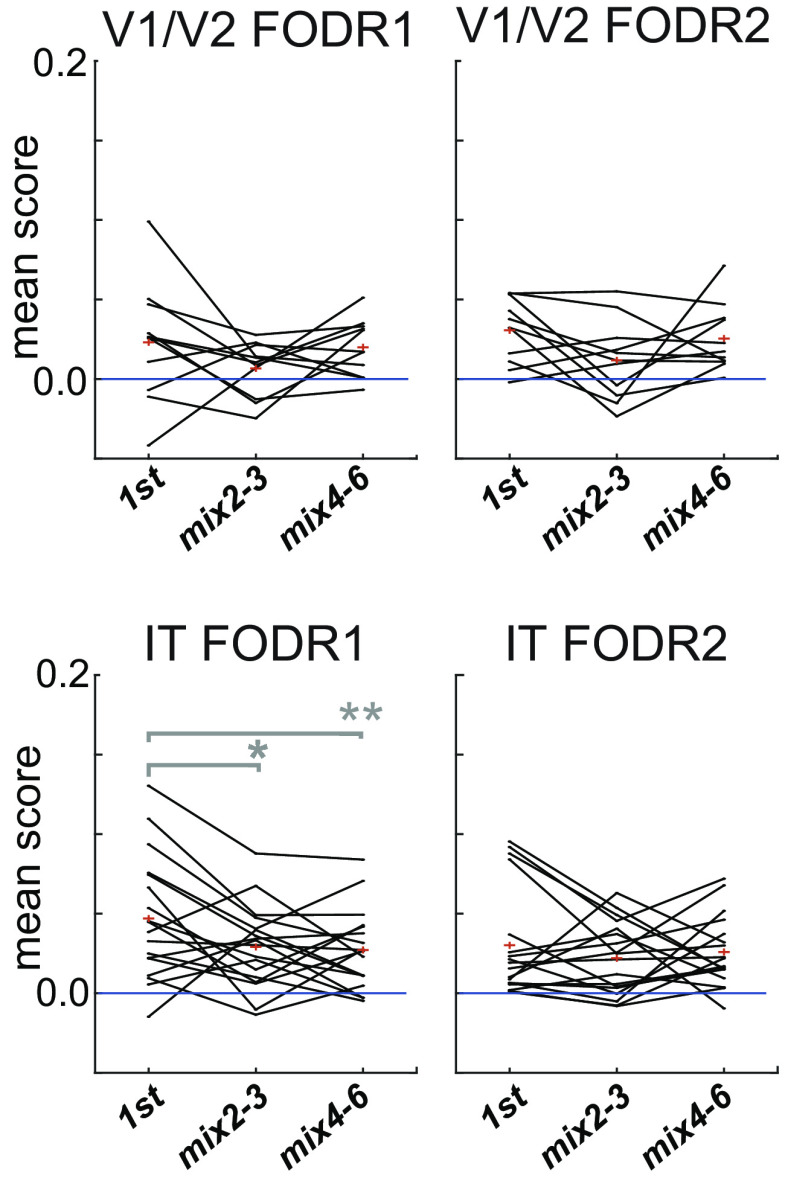
Discrimination accuracy by linear discriminant analysis (LDA) for each area in FODR1 and FODR2. Each line is the 10-fold cross-validation mean score across 10 random selection trials. The chance score calculated by randomization for each session was subtracted [*n* = 11 (V1/V2), 18 (IT)]. The asterisks indicate significant differences compared with first for the two-sided signed-rank test (*p < 0.05, **p < 0.01). The *p*-values are 0.17 (vs. mix2–3) and 0.64 (vs. mix4–6) for V1/V2 of FODR1; 0.083 (vs. mix2–3) and 0.46 (vs. mix4–6), for V1/V2 of FODR2; 0.035 (vs. mix2–3), 0.0065 (vs. mix4–6) for IT of FODR1; and 0.27 (vs. mix2–3), and 0.98 (vs. mix4–6) for IT of FODR2. Extended Data Tables 7-1 and 7-2 summarize the related statistical values.

To study how the behavioral context during free viewing affected neuronal activity, we classified fixations into several categories based on their behavioral implications. First, we classified fixations into object or background fixations. Object fixations were defined as those made within 1.5° from the center of one of the five objects in the scene; all the other fixations were classified as background fixations. We chose the threshold value of 1.5° by considering the object sizes (∼1.0° in radius) and the range of foveal vision (<1.0° in radius). Second, we classified object fixations based on the order of the fixations in the trial. The classification rules differed according to the purpose of the analyses and were as follows: for the analysis shown in Figures 2-4, object fixations were classified into three categories: (1) first fixation: the first fixation on an object after stimulus onset, landing either from another object or background, (2) re-visit fixation: fixation on landing from another object or the background on an object that had already been fixated on in the trial, and (3) second+ fixation: successive fixations on the same object after the first or re-visit fixation. In Figure 5*A*, second+ fixations were further classified into second1 and second2 (and other second+ fixations). Second1 fixations were consecutive fixations just after the first fixation, while second2 fixations were consecutive fixations just after second1 fixations. In Figure 5*B*, re-visit fixations were further classified into re-visit1 and re-visit2 (and other re-visit fixations). Re-visit1 fixations were the first re-visit fixations that occurred in the trial, while re-visit2 fixations were second fixations after re-visit1 fixations. These classifications of the fixation orders were all determined separately for each object. In Figures 5*D*,*E*, 6, and 7, first fixations were the same as described above, but second+ and re-visit fixations were not differentiated. Instead, these later fixations (mix) were classified simply by the number of fixations on the objects in the trial.

10.1523/ENEURO.0086-23.2023.f2-1 Extended Data Figure 2-1 ***A***, Firing rate for the best object. Upper, The population mean firing rate of recorded neurons in each area for first fixations. The colored shaded areas indicate the SEM (*n* = 87, 97, and 337 for V1, V2, and IT, respectively). Lower, Mean firing rate for the best object for first fixation during FODR1. The error bars are the SEM. ***B***, Cumulative plot of fixation durations for each fixation category. Download Figure 2-1, EPS file.

10.1523/ENEURO.0086-23.2023.f3-1 Extended Data Figure 3-1 ***A***, Comparison of sparseness across recording areas. Asterisks indicate significant differences for the two-sided Mann–Whitney *U* test. The *p*-values are 1.8 × 10^−8^ for V2 versus IT in FODR1 and 3.0 × 10^−5^ for V2 versus IT in FODR2. The red lines are the medians and the error bars are the 25th–75th percentiles. ***B***, Decrease in sparseness in second+ and re-visit fixations compared with first fixations during FODR1 and FODR2 for monkey1. The sparseness of first fixations was subtracted for each neuron. Note that recordings that had a minor problem of fixation-saccade identification for technical reasons were not included in the main figure but are shown here. Colors correspond to the fixation orders (*n* = 27, 22, and 96 for V1, V2, and IT, respectively). The white lines are the medians and the error bars are the 25th–75th percentiles. Asterisks indicate statistical significance for the signed-rank test between first fixations and second+ or revisit fixations. The *p*-values are 0.06, 0.004, 0.54, and 0.07 for FODR1-second+, FODR2-second+, FODR1-re-visit, and FODR2-re-visit, for V1; 0.005, 0.002, 0.26, and 0.15 for V2; and 1.4 × 10^−9^, 1.8 × 10^−11^, 1.1 × 10^−6^, and 2.1 × 10^−8^ for IT. ***C***, The same plot as B but for monkey2 (*n* = 80, 93, and 262 for V1, V2, and IT, respectively). The *p*-values are 3.8 × 10^−5^, 4.9 × 10^−7^, 1.7 × 10^−9^, and 3.9 × 10^−9^ for FODR1-second+, FODR2-second+, FODR1-re-visit, and FODR2-re-visit, for V1; 5.7 × 10^−10^, 6.0 × 10^−13^, 1.5 × 10^−12^, and 4.1 × 10^−14^ for V2; and 2.4 × 10^−4^, 2.8 × 10^−11^, 1.0 × 10^−20^, and 3.2 × 10^−27^ for IT. Download Figure 3-1, EPS file.

10.1523/ENEURO.0086-23.2023.tab3-1 Extended Data Table 3-1 Sparseness comparison across fixation order. The *p*-values were determined by the signed-rank test (two sided). The effect size is the Cliff’s δ effect size. Download Table 3-1, DOCX file.

10.1523/ENEURO.0086-23.2023.tab3-2 Extended Data Table 3-2 Sparseness comparison between FODR1 and FODR2. The *p*-values were determined by the signed-rank test (two sided). The effect size is the Cliff’s δ effect size. Download Table 3-2, DOCX file.

10.1523/ENEURO.0086-23.2023.f4-1 Extended Data Figure 4-1 ***A***, Comparison of cosine similarity across recording areas. Asterisks indicate significant differences for the two-sided Mann–Whitney *U* test. The *p*-values are 6.8 × 10^−124^ (V1 vs V2 for FODR1), 2.1 × 10^−74^ (V2 vs IT for FODR1), 2.5 × 10^−140^ (V1 vs V2 for FODR2), and 2.2 × 10^−57^ (V2 vs IT for FODR2). The red lines are the medians and the error bars are the 25th–75th percentiles. ***B***, Decrease in cosine similarity for monkey1. Cosine similarities for first fixations were subtracted from those of second+ and re-visit fixations. Colors correspond to the fixation orders (*n* = 289, 311, and 902 for V1, V2, and IT, respectively). The white lines are the medians and the error bars are the 25th–75th percentiles. Asterisks indicate significant differences for the Kolmogorov–Smirnov test. The *p*-values are 1.6 × 10^−7^ (vs second+ FODR1), 1.4 × 10^−5^ (vs second+ FODR2), 4.1 × 10^−4^ (vs re-visit FODR1), and 9.7 × 10^−5^ (vs re-visit FODR2) for V1; 3.1 × 10^−8^ (vs second+ FODR1), 5.1 × 10^−13^ (vs second+ FODR2), 5.5 × 10^−4^ (vs re-visit FODR1), and 9.6 × 10^−17^ (vs re-visit FODR2) for V2; and 8.7 × 10^−28^ (vs second+ FODR1), 1.4 × 10^−9^ (vs second+ FODR2), 2.2 × 10^−10^ (vs re-visit FODR1), and 1.1 × 10^−9^ (vs re-visit FODR2) for IT. Note that recordings that had a minor problem of fixation-saccade identification for technical reasons were not included in the main figure but are shown here. ***C***, The same plot as A but for monkey2 (*n* = 1000, 1212, and 1552 for V1, V2, and IT, respectively). The *p*-values are 1.1 × 10^−11^ (vs second+ FODR1), 8.1 × 10^−13^ (vs second+ FODR2), 2.9 × 10^−5^ (vs re-visit FODR1), and 5.7 × 10^−15^ (vs re-visit FODR2) for V1; 5.1 × 10^−12^ (vs second+ FODR1), 1.8 × 10^−62^ (vs second+ FODR2), 2.0 × 10^−7^ (vs re-visit FODR1), and 3.1 × 10^−19^ (vs re-visit FODR2) for V2; and 2.8 × 10^−49^ (vs second+ FODR1), 3.1 × 10^−81^ (vs second+ FODR2), 1.3 × 10^−36^ (vs re-visit FODR1), and 7.4 × 10^−74^ (vs re-visit FODR2) for IT. ***D***, Simulation to examine the effect of a firing rate decrease. Cosine similarities for simulated first fixations were subtracted from those of simulated second+ and re-visit fixations. The reductions for later fixations were 0.80 (second+ FODR1), 0.83 (re-visit FODR2), 0.75 (second+ FODR2), and 0.84 (re-visit FODR2) for V1; 0.77 (second+ FODR1), 0.85 (re-visit FODR2), 0.68(second+ FODR2), and 0.85 (re-visit FODR2) for V2; and 0.72 (second+ FODR1), 0.71 (re-visit FODR2), 0.78 (second+ FODR2), and 0.74 (re-visit FODR2) for IT. Colors correspond to the fixation orders (*n* = 1014, 1245, and 2529 for V1, V2, and IT, respectively). The white lines are the medians, and the error bars are the 25th–75th percentiles. Asterisks indicate a significant difference for the Kolmogorov–Smirnov test. The *p*-values are 0.10 (vs second+ FODR1), 0.12 (vs second+ FODR2), 0.50 (vs re-visit FODR1), and 0.65 (vs re-visit FODR2) for V1; 0.047 (vs second+ FODR1), 2.1 × 10^−5^ (vs second+ FODR2), 0.058 (vs re-visit FODR1), and 0.0065 (vs re-visit FODR2) for V2; and 5.2 × 10^−6^ (vs second+ FODR1), 7.6 × 10^−8^ (vs second+ FODR2), 1.6 × 10^−4^ (vs re-visit FODR1), and 3.9 × 10^−8^ (vs re-visit FODR2) for IT. Download Figure 4-1, EPS file.

10.1523/ENEURO.0086-23.2023.tab4-1 Extended Data Table 4-1 Comparison of cosine similarities across fixation orders. The *p*-values were determined by the Kolmogorov–Smirnov test (two sided). The effect size is the Cliff’s δ effect size. Download Table 4-1, DOCX file.

10.1523/ENEURO.0086-23.2023.tab4-2 Extended Data Table 4-2 Comparison of cosine similarities between FODR1 and FODR2. The *p*-values were determined by the Kolmogorov–Smirnov test (two sided). The effect size is the Cliff’s δ effect size. Download Table 4-2, DOCX file.

10.1523/ENEURO.0086-23.2023.f5-1 Extended Data Figure 5-1 ***A***, Comparison of the firing rates of different presentation order categories in the fixation task. The population mean firing rates of recorded neurons in each area. Colors indicate different presentation order categories. Colored shaded areas indicate the SEM (*n* = 723, 1,100, and 1,569 for V1, V2, and IT, respectively). Asterisks indicate the time points that showed a significant difference in mean firing rates between first and other presentation orders (signed-rank test, *p *<* *0.01, Bonferroni corrected). Light blue and yellow shaded areas indicate periods 1 and 2, respectively. ***B***, Comparison of cosine similarities among presentation orders. The black lines are the mean cosine similarities (*n* = 1,647, 1,729, and 1,281 for V1, V2, and IT, respectively). The red lines are the medians and the error bars are the 25th–75th percentiles. Asterisks indicate significant differences for the two-sided Kolmogorov–Smirnov test. The *p*-values are 0.25 (vs second), 0.016 (vs third), 0.13 (vs fourth), and 0.0056 (vs 5th) for V1 period1; 2.6 × 10^−4^ (vs second), 0.024 (vs third), 7.3 × 10^−6^ (vs fourth), and 0.070 (vs fifth) for V1 period2; 0.024 (vs second), 0.43 (vs third), 0.82 (vs fourth), and 0.18 (vs fifth) for V2 period1; 0.49 (vs second), 0.0036 (vs third), 0.41(vs fourth), and 0.0022 (vs fifth) for V2 period2; 0.17 (vs second), 0.00064 (vs third), 1.3 × 10^−5^ (vs fourth), and 0.0029 (vs fifth) for IT period1; and 1.1 × 10^−5^ (vs second), 0.00014 (vs third), 0.0012 (vs fourth), and 1.2 × 10^−06^ (vs fifth) for IT period2. Green lines are the results of linear fitting to the mean cosine similarities across fixation orders. The slopes are −0.003 (V1 Period1), 4.7 × 10^−4^ (V1 Period2), −0.0065 (V2 Period1), −0.005026 (V2 Period2), 0.0022 (IT Period1), and 1.2 × 10^−6^ (IT Period2). Download Figure 5-1, EPS file.

10.1523/ENEURO.0086-23.2023.tab5-1 Extended Data Table 5-1 Comparison of cosine similarities between second1 and second2 fixations. *p*-values were determined by the s Kolmogorov–Smirnov test (two sided). The effect size is the Cliff’s δ effect size. Download Table 5-1, DOCX file.

10.1523/ENEURO.0086-23.2023.tab5-2 Extended Data Table 5-2 Comparison of cosine similarities between re-visit1 and re-visit2 fixations. *p*-values were determined by the Kolmogorov–Smirnov test (two sided). The effect size is the Cliff’s δ effect size. (h) indicates that mean2 is higher than mean1. Download Table 5-2, DOCX file.

10.1523/ENEURO.0086-23.2023.tab5-3 Extended Data Table 5-3 Comparison of cosine similarities across saccade order, first, and mix second to fifth fixations. *p*-values were determined by the Kolmogorov–Smirnov test (two sided). The effect size is the Cliff’s δ effect size. Download Table 5-3, DOCX file.

10.1523/ENEURO.0086-23.2023.tab5-4 Extended Data Table 5-4 Slopes of the mean cosine similarities by linear least-square fitting. Download Table 5-4, DOCX file.

10.1523/ENEURO.0086-23.2023.tab5-5 Extended Data Table 5-5 Comparison of cosine similarities between FODR1 and FODR2. The *p*-values were determined by the Kolmogorov–Smirnov test (two sided). The effect size is the Cliff’s δ effect size. Download Table 5-5, DOCX file.

10.1523/ENEURO.0086-23.2023.tab6-1 Extended Data Table 6-1 Comparison of ROC between saccade orders. The *p*-values were determined by the signed-rank test (two sided). The effect size is the Cliff’s δ effect size. Download Table 6-1, DOCX file.

10.1523/ENEURO.0086-23.2023.tab6-2 Extended Data Table 6-2 Comparison of ROC between saccade orders. The *p*-values were determined by the signed-rank test (two sided). The effect size is the Cliff’s δ effect size. Download Table 6-2, DOCX file.

10.1523/ENEURO.0086-23.2023.tab7-1 Extended Data Table 7-1 Comparison of discrimination accuracy by LDA between first and later fixations. The *p*-values were determined by the signed-rank test (two sided). The effect size is the Cliff’s δ effect size. Download Table 7-1, DOCX file.

10.1523/ENEURO.0086-23.2023.tab7-2 Extended Data Table 7-2 Comparison of discrimination accuracy by LDA between FODR1 and FODR2. The *p*-values were determined by the signed-rank test (two sided). The effect size is the Cliff’s δ effect size. Download Table 7-2, DOCX file.

#### Overlap of fixation positions

To estimate the similarity in fixation positions between different fixation categories, we created a smooth two-dimensional (2D) probability distribution (similar to a fixation position heat map), which indicated the fixation occurrences within an object stimulus. We created the distribution by summing multiple 2D Gaussian functions wherein means were the relative positions of each fixation from the center of each object and whose variances were 0.2°. The resulting distribution was normalized to a maximum value of 1. We derived these fixation probability distributions from the fixations for each object and each fixation category separately. The overlap value for an object was calculated by comparing two fixation probabilities of different fixation categories of the same object. Specifically, the overlap value was calculated by dividing the sum of the overlap of two distributions by the total (i.e., two). For example, if the fixation probability functions were identical, the overlap value was 1.

#### Spike sorting and spike-train segmentation

The extracellular action potentials, or spikes, were primarily processed using the Kaneko–Tamura–Suzuki (KTS) spike sorting algorithm (Kaneko et al., 2007; Tamura et al., 2014). The KTS sorter uses the signals of multiple electrode channels to track gradual changes in the spike waveforms over time. We used the KTS spike sorting results to extract neural firing timings during the fixation tasks. Unfortunately, for free viewing, because of the geometry of our linear electrode arrays, spike waveforms were only detected simultaneously in one or two electrode channels. In addition, the long durations of our recordings (>30 min), which were beyond the expectation of the KTS algorithm (<10 min), amplified the unwanted effects of electrode drift, such as gradual/abrupt changes in the spike amplitudes over time and gradual/abrupt changes in the firing rates of sorted units. Further inspection of the resultant single-unit spike-trains, which we termed KTS units, revealed that some of them had been contaminated with spikes belonging to other units. Additional postprocessing was therefore necessary to improve the sorting of these KTS units to reliably extract neuron firing data during the free viewing tasks. The postprocessing comprised the following steps: (1) spike extraction: the spike waveforms of the KTS units were extracted from the continuous raw signals (sampled at 20 kHz) by applying a high-pass filter at 500 Hz. The obtained waveforms were up-sampled to 200 kHz via cubic interpolation and aligned with their waveform minima; (2) re-clustering: we applied principal component analysis to the spike waveforms of each KTS unit, and the spikes were re-clustered up to three clusters in a space spanned by the first three principal components using the Gaussian Mixture Model method. These clusters were the preliminary single-unit activity (SUA). We rejected clusters with a refractory period violation (threshold at 1.2 ms) of their interspike intervals of above 0.1% to avoid multiunit contamination; (3) cluster merging: initial SUA spikes belonging to an identical unit could be separated into multiple clusters. To avoid over-disaggregation of SUA, pairwise cross-correlations between cluster-average spike waveforms were computed for all pairs of clusters. Pairs of clusters with *R*^2^ > 0.95 were merged into one cluster. Three independent human inspectors checked the validity of each cluster merging, and the merging was approved when all the three inspectors confirmed the validity.

#### Perifixation time histogram (PFTH)

For the presentation of the PFTHs in Figure 2, spikes around the fixation onset were collected for each fixation and binned into 1 ms. These binned spike counts for each fixation were smoothed using a Gaussian kernel with a SD of 10 ms to create a spike density function for each fixation. After smoothing, they were averaged across fixations.

#### Determination of the fixation order-dependent response (FODR) periods

The FODR time windows were determined by comparing the number of neurons that showed a significant decrease in firing rates (Mann–Whitney *U* test, *p *<* *0.01) in the later fixations, compared with the first fixations, across different time windows within 0–200 ms of fixation onset. We included all fixations, regardless of object identity, for this calculation. The number of neurons with significant firing decreases showed two peaks in time in all three visual areas; we selected the time range for FODR1 and FODR2 as the time range centered on these two peaks.

#### Sparseness

Object selectivity sparseness for an individual neuron was defined as

$$S_{life}=\frac{1-a}{1-\frac{1}{N}},a=\frac{{\left(\left(\sum r_{i}\right)/N\right)}^{2}}{\sum \left(r_{i}^{2}/N\right)},$$

where *r_i_* was the response firing rate of a neuron to the *i-*th object, and *N* was the total number of presented objects during the recording of this neuron (Rolls and Tovee, 1995a). Here, the “original” sparseness value, *a,* was converted to a normalized sparseness value (*S_life_*) between 0 (dense) and 1 (highly sparse). For a fair comparison between different fixation categories, the sparseness for each neuron was calculated using only the reduced response vectors that contained valid mean firing rates for all three fixation categories.

#### Cosine similarity

To quantify (dis)similarities in population activity in response to different objects, we defined a population response vector for each object, composed of the mean firing rates of individual neurons in response to the object. First, the number of firings, *r_i_*, for neuron *i* was separately pooled for each of the fixation categories and objects fixated on. The objects that had at least 10 samples for all fixation categories were used for the analysis. To calculate cosine similarity between objects *j* and *j'*, corresponding firings *r_j_* and *r_j'_* were pulled randomly from the pool for all the available neurons. Then, the population response vector, *R_j_*, for object j, was determined by *R_j_ = (r_1j_, r_2j_, …r_Nj_)*, where *N* was the total number of neurons. If the number of available neurons was less than five, the object was not used for the calculation. The cosine similarity, *Sim_jj’_*, between the population response vectors *R_j_* and *R_j'_*, for objects j and j', was calculated with the following formula:

$$Sim_{jj^{\text{'}}}=\cos\left(\theta_{jj\text{'}}\right)=\frac{R_{j}R_{j\text{'}}}{\left|R_{j}\right|\left|R_{j\text{'}}\right|},$$

where *θ_j j'_* was the angle between *R_j_* and *R_j'_.* Note that because firing rates can only be positive values, the similarity value will be between 0 and 1, where the value of 0 means “orthogonal” population responses to the two objects and the value of 1 means identical responses. To determine single *Sim_jj'_*, random sampling was performed 100 times and a mean value was calculated. Cosine similarities between pairs of vectors in a space is not independent. However, our analysis on cosine similarity is focused on comparison of the similarity values for responses to a pair of objects in two different fixation conditions. Thus, statistical tests are preformed on the differences between the similarity values for the same object pair in different fixation conditions. Our null hypothesis is that the change in population response to an object from one fixation condition to another is independent between different objects, and hence the cosine similarity between population responses to a pair of objects changes randomly across fixation conditions. We believe that there are no a priori reasons to assume systematic dependencies between changes in these cosine similarities across conditions, although the responses to the same object pair (in different conditions) are considered for comparison.

#### Singular value decomposition

Only the recording sessions that had more than five simultaneously recorded neurons and >10 fixated objects for all fixation categories served for this analysis. Five, 10, and 18 sessions for V1, V2, and IT, respectively, met these criteria. For each session, a population response matrix *R*, for each fixation category, was constructed with the response firing rates r_ij_ of neuron j and object i belonging to the session as its elements. The matrix *R*’s singular value decomposed as

$$R=UDV^{T}=\sum_{k}d_{k}u_{k}v_{k}^{T},$$

where d_k_ were the singular values, column vectors u_k_ represented object tuning profiles, and column vectors v_k_ represented neural population activity profiles. The diagonal elements, u_k_, of the resulting matrix *D* were the relative weights for the components of the original matrix *R* represented by cross products of the columns u_k_ and v_k_, of *U* and *V*, respectively. The singular values were plotted cumulatively, and the area under the curve (AUC) was calculated for each plot. The AUC quantifies the un-uniformity of the singular values; when all the singular values are the same, AUC equals 0.5. As the uniformity of the values decreases, AUC increases toward 1, which corresponds to the case where only one singular value is non-zero.

#### Linear discriminant analysis (LDA)

As the number of simultaneously recorded neurons was generally small (less than five) for V1 and V2, we combined the neural data for these two areas. In addition, since the number of fixations for an object decreased as the fixation order increased, we pooled the data for mix2 and mix3 fixations together, and mix4, mix5, and mix6 fixations together, to obtain sufficient inputs for the discrimination model. Only data from the recording sessions that had more than five simultaneously recorded neurons and >10 fixated objects were used for this analysis. Furthermore, each of the objects had to fulfill the criterion that the fixation numbers of all three fixation categories were >10. For fairness, the number of input samples was kept the same across the three fixation categories; we randomly pulled the same number of samples from each fixation category. Scores (proportions correctly classified) were calculated using 10-fold cross-validation predictions. The chance level scores were calculated by randomizing the correspondence between the object identification labels and the neural response for each set of samples. We used the “fitcdiscr” function in Statistics and the machine learning toolbox of MATLAB (MathWorks) for these calculations.

## Results

### Eye movement behavior during free viewing

We trained two macaque monkeys (1 and 2) to perform a free viewing task (Fig. 1*A*). The task required the monkeys to freely view a random array of object images during a 5-s presentation of a stimulus image to obtain their reward. Each stimulus image consisted of a gray background with five different (colored) objects placed at random positions (for details, see Materials and Methods, Behavioral tasks and Visual stimuli for free viewing). Although the monkeys were not explicitly trained to fixate on the embedded objects, they nonetheless mainly fixated on the objects. Figure 1*B* shows an example trace of eye movement trajectories (black line) and fixation positions (red dots) overlaid on the presented stimulus image. Here, the monkey moved its gaze from one object to another, repeatedly fixated on the same object, and visited the same object after fixations on another object. This was typical behavior, observed across most of the recordings.

We first determined the periods of fixations and saccades (see Materials and Methods, Data analysis and Detection of fixations and saccades) and then classified each fixation as either object fixation (within 1.5° of the object center) or background fixation (farther than 1.5° of the object center). Ninety percent of the identified fixations were object fixations. We further classified the object fixations into three categories: first, re-visit, and second+ fixations (Fig. 1*C*), based on the order of the fixations within a trial. If a monkey fixated on all five objects in an image, five out of all fixations in this trial were registered as first fixations. Further object fixations were classified into second+ fixations, which were subsequent and consecutive fixations on the same object, or re-visit fixations, which were fixations on an object that had already been fixated on within the same trial.

This categorization allowed for differentiation between large and small changes in visual inputs caused by eye movements; the first and re-visit fixations were preceded by saccades from one object (or background) to another, causing a large change in visual inputs, whereas second+ fixations were confined within the regions of objects already being fixated on, causing a relatively small change in visual inputs. As shown in Figure 1*D*, retinal shifts (saccade amplitude) were in fact larger for the first and re-visit fixations compared with the second+ fixations (mean ± SD: 8.9 ± 5.1°, 0.9 ± 0.4°, and 7.6 ± 5.0° for first, second+, and re-visits, respectively, Mann–Whitney *U* test, *p *<* *0.01; data from all fixations of both monkeys). The statistical analyses for these results are summarized in Extended Data Table 1-1. The fixation position distributions were more similar between the first and re-visit fixations compared with those of second+ and other fixations (Fig. 1*E* shows example fixation positions). We calculated the overlap of fixation positions for each object (see Materials and Methods for computation of fixation position overlap). As can be seen from the distribution of overlap values in Figure 1*F*, larger overlaps were calculated between the first and re-visit fixations compared with those between the first and second+ or between re-visit and second+ fixations (mean: 0.88, 0.75, and 0.76 for first vs. re-visit, first vs. second+, and re-visit vs. second+, respectively, signed-rank test *p *<* *0.01). The statistical analysis for these results is summarized in Extended Data Table 1-2. These results show that the retinal inputs were more similar between the first and re-visit fixations than those between the second+ fixations and other fixations. In addition, only the second+ fixations had the same object fixation as the preceding fixation. In contrast, the second+ and re-visit fixations had a commonality in terms of behavioral context; both fixations occurred when the monkey was already fixated on a previously fixated object. Comparisons of neuronal activity during these different fixation conditions allowed us to study the effect of the behavioral context while viewing the same object.

### After the first fixation, the firing rate decreased in later fixations

We extracted the single-unit spiking activity of V1, V2, and IT neurons (*n* = 87, 97, and 337, respectively). Figure 2*A* shows the firing rate modulations of example V1, V2, and IT neurons as PFTHs, with the data aligned on fixation onset (black line) and regardless of the identity of the object fixated on or the fixation category. Typically, V1 and V2 neurons showed a slight decrease in the firing rate around 30 ms after fixation onset (the mean and SD of trough latency for V1 was 28.5 ± 11.9 ms, *n* = 32, and those for V2 were 41.6 ± 41.4 ms, *n* = 27), followed by a substantial excitatory response approximately 50–80 ms (blue line) after fixation onset (mean and SD of trough latency for V1 was 66.9 ± 42.6 ms, *n* = 59 and those for V2 were 77.3 ± 44.9 ms, *n* = 61). This excitatory response was occasionally followed by delayed activity approximately 130 ms after fixation onset, which was prominent in the example V2 neuron. In comparison, IT neurons typically showed weak or no decrease in firing rate and a later broader peak, approximately 100 ms after fixation onset.

To compare the firing rates between fixation categories, we constructed separate population PFTHs for the three categories (first, second+, and re-visits are shown in Fig. 1*C*; Fig. 2*B*). The firing rates of first fixations were higher than those of second+ or re-visit fixations at approximately 40–150 ms (V1 and V2) or 90–200 ms (IT) after fixation onset. A reason for the modulations caused by object fixations being small is because it includes all fixations as samples, regardless of the fixated object. Preferable objects for the neurons evoked much higher spiking rates (Extended Data Fig. 2-1*A*). The asterisks in Figure 2*B* indicate the periods in which the first fixations had a significantly higher firing rate (*p *<* *0.01) than second+ or re-visit fixations, from 0 to 200 ms from fixation onset (signed-rank test, bin size: 10 ms, *n* = 87, 92, and 337 for V1, V2, and IT, respectively). In the statistical analysis, we considered the assessed time interval between 0 and 200 ms after fixation onset to avoid contamination of activity modulations by subsequent saccades and fixations (Extended Data Fig. 2-1*B*; for 50% of first fixations, subsequent saccades started at 200 ms). For V1 and V2 in Figure 2*B*, asterisks are shown for two separate time ranges: an initial range corresponding to the first sharp peak and a second range corresponding to a later, moderate decay in firing rates. This separation of the time range was not evident for IT. To quantify this observation, we performed significance tests for differences in firing rates for each neuron on the first and later (second+ and re-visit together) fixations in a sliding window manner (Mann–Whitney *U* test, *p *<* *0.01). In the plots for V1 and V2 in Figure 2*C*, which show the temporal change in the number of neurons that displayed significant differences, two peaks are evident. For IT, the separation of the peaks is less clear than for V1 and V2. However, for a fair comparison across the areas, we selected, for each area, the time range corresponding to each of the two peaks (shown in light blue and light yellow). We named the obtained time ranges FODR periods 1 and 2. FODR1 for V1, V2, and IT were 40–90, 40–90, and 100–150 ms, respectively, while FODR2 were 110–160, 110–160, and 150–200 ms, respectively. Further analyses were focused on the spike rates during these FODR periods.

### Neurons became more selective in later fixations than in first fixations

In the previous section, we examined the dependence of the mean firing rates only on the fixation order, regardless of the identity of the objects fixated on. To include object identities in the analysis, we calculated separate mean firing rates within the FODR periods for fixations on each object. Figure 3*A* shows the dependence of firing rate on object identity, or in other words, object selectivity for representative neurons in V1, V2, and IT. In these examples, the firing rates generally decreased for the later (second+ and re-visit) fixations [spikes/s (mean ± SD): 24.2 ± 7.4 for first, 15.8 ± 6.7 for second+, and 17.1 ± 7.3 for re-visit in the V1 example; 15.1 ± 5.1 for first, 7.5 ± 4.8 for second+, and 9.5 ± 4.9 for re-visit in the V2 example; and 10.1 ± 6.6 for first, 4.9 ± 4.3 for second+, and 5.5 ± 6.9 for re-visit in the IT example].

Despite this general trend, the response to each individual object did not necessarily decrease and there were a small number of instances where the response increased in later fixations. To quantify this observation, we counted the number of objects that caused either increased or decreased firing rates from the first to later fixations for each neuron. Figure 3*B* shows the joint distribution of these counts across neurons. For most neurons, the number of objects that caused decreased firing rates in later fixations was greater than those that caused increased firing rates, which is consistent with our previous observation (Fig. 2). We examined the number of objects that significantly (Mann–Whitney *U* test, corrected with false discovery rate *p *<* *0.05) increased or decreased in later fixations. The number of neurons that had an increased response to more than one significant object in later fixations were 2/55, 2/55, 2/67, 1/67, 7/230, and 7/230 for V1 FODR1, V1 FODR2, V2 FODR1, V2 FODR2, IT FODR1, and IT FODR2, respectively. This number was smaller than the number of neurons that had a decreased response to more than one significant object in later fixations (23/55, 26/55, 27/67, 23/67, 64/230, and 62/230 for V1 FODR1, V1 FODR2, V2 FODR1, V2 FODR2, IT FODR1, and IT FODR2, respectively).

The observed response modulation indicated that the object selectivity of each neuron was enhanced in later fixations. To directly quantify object selectivity, we calculated the object selectivity sparseness of each neuron’s responses separately for each fixation category. Sparseness is a commonly used measure for object selectivity (Rolls and Tovee, 1995a; Vinje and Gallant, 2000; Lehky et al., 2005), with a large value indicating that the neuron responds to a small number of stimuli (see Materials and Methods for formulation), and therefore the neuron’s response is more selective to a particular set of stimuli. The respective object selectivity sparseness values of the example neurons shown in Figure 3*A* for first, second+, and re-visit fixations were determined to be 0.09, 0.16, and 0.15 for V1; 0.10, 0.29, and 0.22 for V2; and 0.30, 0.45, and 0.58 for IT, indicating enhanced sparseness of these neurons in later fixations.

The sparseness values followed the trend V1 ≤ V2 < IT (mean: 0.38, 0.39, and 0.54 for V1, V2, and IT in FODR1, and 0.43, 0.45, and 0.55 for V1, V2, and IT in FODR2; Extended Data Fig. 3-1*A*). This observation is consistent with the widely accepted view that representation becomes sparser as one ascends higher in visual hierarchy (Barlow, 1972). The high sparseness can also be explained by the nature of IT neurons, which show strong selectivity for objects but not for low-level visual features. The comparison between our fixation categories showed a consistent increase in sparseness in later fixations (second+ and re-visit) in all visual areas during both FODR periods (Fig. 3*C*; Extended Data Table 3-1). A similar tendency was observed for the individual animals (Extended Data Fig. 3-1*B*,*C*). Statistically significant differences between second+ and re-visit fixations in V1 and IT were also observed (signed-rank test *p *<* *0.01), although the effect sizes were much smaller than those between first and later fixations. While comparisons between FODR1 and FODR2 in the same fixation categories showed increased sparseness in FODR2 compared with FODR1 (Extended Data Table 3-2), the sparseness increase was relatively mild. Overall, during free viewing, the object selectivity of individual neurons became sharper in later fixations (second+ and re-visit), and there was a tendency for later responses to become sparser from an earlier period (FODR1) to a later period (FODR2) within single fixations.

### Population activity patterns were dissimilar between different objects in later fixations

We then sought to determine how sharpening of the object selectivity of individual neurons within a trial affected information coding at the population level. To quantify the (dis)similarities in population activities in response to different objects, we defined for each object a population response vector composed of the mean firing rates of individual neurons in response to the object. To quantify the differences between the population responses, we calculated the cosine similarity between every possible pair of population response vectors for the different objects. The cosine similarity equaled one when the two vectors were in the same direction, i.e., their associated population activities were identical up to a scaling factor, and it equaled 0 when the vectors were orthogonal to each other, i.e., the two population activities were maximally different. For the example population response patterns of the two objects in Figure 4*A*, the cosine similarity between the pairs (shown at the top of each histogram) was higher for first fixations than later fixations, and this difference was more prominent in the higher visual area (IT) than in the lower areas (V1 < V2 < IT). Figure 4*B* shows the distribution of cosine similarity values for all possible combinations of objects (*n* = 991, 1210, and 2342 for V1, V2, and IT, respectively), calculated separately for each fixation category [first (red), second+ (blue), and re-visit (purple)] for each FODR period. The distribution of cosine similarity values is skewed toward larger values (closer to 1) for V1 compared with the distributions of V2 and IT, which showed more prominent dissimilarities (means: 0.61, 0.44, and 0.34 for V1, V2, and IT, respectively, for FODR1 and 0.54, 0.38, and 0.31, for V1, V2, and IT, respectively, for FODR2). V1 and V2 or V2 and IT showed significant differences in the Mann–Whitney *U* test for both FODR1 and FODR2 *(p *=* *6.8 × 10^−124^ and 2.1 × 10^−74^ for V1-V2 and V2-IT, respectively, in FODR1 and 2.5 × 10^−140^ and 2.2 × 10^−57^ for V1-V2 and V2-IT, respectively, in FODR2; Extended Data Fig. 4-1*A*). Comparisons between the fixation categories revealed weaker similarities in later fixations than the first fixations in all three areas for both FODR periods (Fig. 4*C*; Extended Data Table 4-1), indicating that object representation by the neural population was more distinct in later fixations than in the first fixations in these areas. A similar tendency was observed for the individual animals (Extended Data Fig. 4-1*B*,*C*). While there was also a significant difference between second+ and re-visit fixations, the effect size was much smaller than that between first and later (second+ and re-visit) fixations (Extended Data Table 4-1). In the comparison between FODR1 and FODR2, we observed a significant decrease in similarity in FODR2 (Extended Data Table 4-2), although the effect size was intermediate or minor. To examine the impact of firing rate reduction on cosine similarity, we compared the cosine similarity calculated using firing rates extracted from a Poisson distribution based on the mean firing rates for each object during the first fixation with those calculated using firing rates from a Poisson distribution that was reduced by the same amount of mean firing rates in later fixations (Extended Data Fig. 4-1*D*). While simulations in second+ fixations of V2, and both second+ and re-visit fixations in IT, showed statistically significant decreases in cosine similarity, all the effect sizes were smaller than the actual results, suggesting that the observed decrease in cosine similarity cannot be solely explained by firing rate reduction alone.

### Closer examination of the effects of the fixation order

In the previous sections, we observed increased sparseness and decreased cosine similarity in later (second+ and re-visit) fixations compared with the first fixations, emphasizing clear differences between first and later fixations. We used this categorization because we initially considered that neural activity could be more similar between first and re-visit fixation than between first and second+ fixations because the retinal inputs were more similar between first and re-visit fixations than between first and second+ fixations. However, we did not detect noticeable differences in neural activity between second+ and re-visit fixations. In this section, we hypothesize that neuronal population activity may be affected by the number of experiences of the objects in the trials.

To examine the effect of the number of consecutive fixations in second+ fixations in a trial, we evaluated the first two second+ fixations, denoted here as second1 (the first second+ fixation, meaning the second of the consecutive fixations on the same object) and second2 (the subsequent second+ fixation, meaning the third of the consecutive fixations on the same object), to determine whether the dissimilarity continuously developed across two consecutive second+ fixations.

As shown in Figure 5*A* and Extended Data Table 5-1, the second2 similarity was lower than the second1 similarity in all three visual areas for both FODR1 and FODR2, indicating an increase in dissimilarity across consecutive second+ fixations. In a similar manner, we extracted corresponding values for re-visit1 (first fixation among re-visit fixations) and re-visit2 (second fixation among re-visit fixations) from all re-visit fixations. We found that the cosine similarity did not decrease between re-visit1 and re-visit2 (Fig. 5*B*; Extended Data Table 5-2).

This may have occurred because the similarity values for re-visit1 were already low and therefore did not decrease further for re-visit2. Also note that re-visit1 and re-visit2 were not consecutive and there may have been second+ fixations between re-visit1 and re-visit2. Figure 5*C* shows the change in the number of fixations within single trials. As expected, the number of second1 fixations started to increase earlier than the re-visit1 fixations. Given these observations, we hypothesized that the cosine similarity decrease was associated simply with the number of previous fixations in a trial (regardless of whether they were second or re-visit). To test this, we reclassified the fixations into first (same as the previous classifications) and mix2, mix3, and others, where the classification was simply based on the accumulated fixation number for each object (Fig. 5*D*). The cosine similarity gradually decreased across accumulated fixation numbers (Fig. 5*E*; Extended Data Table 5-3), and the slopes of the mean cosine similarities across the fixation orders showed negative values in all areas (Fig. 5*E*, red line plot; Extended Data Table 5-4). The comparison of FODR1 and FODR2 (Extended Data Table 5-5) showed a decrease in similarity in most cases in all visual areas. However, the effect size was relatively small. Overall, our results suggest that the accumulation of fixation experience gradually decreases the similarity of population activity in response to different objects. Furthermore, a slight reduction in similarity also occurs even within a single fixation. To compare the observed reduction in cosine similarity across fixations, we performed the same analysis for the responses of neurons in conventional fixation tasks for the same monkey (Extended Data Fig. 5-1). In this case, the order of the object stimuli was randomized, and the duration of stimulus presentation was fixed at 200 ms with 200-ms interstimulus intervals. The cosine similarity decreased slightly in later stimulus presentations. However, the magnitude of the decrease did not increase as the presentation order increased, as was seen in free viewing (Fig. 5*E*). The slope of the mean cosine similarity across the presentation order was around 0, which differed markedly from those calculated in the free viewing analyses.

### Object representation becomes complex in later fixations

Cosine similarity is a pairwise comparison of the population activity between two objects. To examine the population profiles of more than two objects, we performed singular value decomposition (SVD) for the firing rate matrix *R* (object ID × neuron ID; see Materials and Methods for the generation of *R*). We evaluated the complexity of the matrix *R* by comparing the singular value vectors which represents the weights of the individual components (Fig. 6*A*).

$$R={\text{ UDV}}^{\text{T}}.$$

SVD decomposes a matrix into a weighted sum of outer products of column vectors (from *U*) and row vectors (from *V^T^*). In the present case, we can interpret each column vector of *U* as an object tuning profile of a neuron and the corresponding row vector of *V^T^* (or column vector of *V*) as an indicator of how much different neurons contribute to the representation of an object. The components of the diagonal matrix *D*, i.e., the singular values, represent the degrees of influence of the object tuning profiles on neuronal activity.

If we assume that there are only a small number of large singular values while many others are close to zero the matrix is simple and we can precisely reconstruct it by a reduced number of components, i.e., a slight variation in activity profiles. If many singular values are non-zero, the matrix is complex. Many components are then needed to reproduce the pattern. We derived the normalized cumulative singular values to evaluate the differences between fixation orders (Fig. 6*B*) and compared the AUCs.

We created the matrix *R* for each recording session separately for each fixation category and calculated the AUC values of each matrix *R*. Figure 6*C* shows the comparison of AUC values between fixation orders. The result shows a gradual decrease in mean AUC and an increase in effect size (Extended Data Table 6-1, signed-rank test *p *<* *0.05) in all areas (although there was no statistically significant difference in V1, probably because of the small number of samples). This decrease in AUC was observed in both FODR1 and FODR2. In contrast, the mean comparison between FODR1 and FODR2 showed a slight reduction in AUC values only in IT, but there were no statistically significant results (Extended Data Table 6-2). This mild effect is consistent with the results of the cosine similarity analysis.

### Examination of object discrimination accuracy by population response

Having shown a decrease in the similarity of population responses to different objects across consecutive fixation orders, we then sought to infer object identity from population activity. We performed LDA on simultaneously recorded spiking activities and examined whether an object’s discriminability by population activity changed across fixation orders. Since the number of samples became fewer across consecutive fixations, we pooled mix2 and mix3 together in one group (mix2–3), and mix4, mix5, and mix6 in another group (mix4–6). The same number of input data samples for LDA were randomly picked up from the pool of each group.

As can be seen in Figure 7, which shows the 10-fold cross-validation mean scores for 10 repetitions of the random pick-up (chance level subtracted), most of the traces were above 0, meaning that population activity discriminated the objects better than would be expected by chance. It should be noted that the number of simultaneously recorded units was mostly less than the number of objects to discriminate, making this classification task highly demanding. There were no significant differences in the mean scores between the first and either the mix2–3 or mix4–6 groups in either FODR1 or FODR2 for V1/V2 (Fig. 7; Extended Data Table 7-1). For IT, there was a slight decrease in the mean scores in later fixations in FODR1. The comparison between FODR1 and FODR2 showed a significant difference only in IT for the first fixations (Extended Data Table 7-2). In summary, we conclude that the discriminability of objects from simultaneously recorded neural populations does not differ, or slightly decreases, across fixation orders. We discuss these results of LDA, in relation to cosine similarity and sparseness, in the discussion section.

## Discussion

We studied the changes in individual and population neuronal activity in three different visual areas, V1, V2, and IT, of macaque monkeys during free viewing of random arrays of object images. The animals freely selected the objects in the stimulus image to fixate on. We focused on the relationship between spiking activity, the order of fixations, and the identity of the fixated objects, and found a reduction in mean firing rates in later fixations compared with the first fixations in all recorded areas. The responses of individual neurons became sparser, and thus more selective, for individual objects, and the population representation of objects became distinct between the objects over repeated fixations. Moreover, we found that activity became slightly sparser and more selective in later periods, even within a fixation. These results suggest that visual neuronal activity is dynamically modulated depending on sensory experiences, and that a smaller number of spikes allows for the discrimination of individual objects in a scene. These changes in neural activity across fixations during free viewing were not fully reproduced by giving the same set of object stimuli in a random order, passively, in fixation tasks.

We found that the visual experience dynamically affected visual neuronal activity to produce a population response that was beneficial for object discrimination and that experience-dependent modulation of visual neuronal activity can occur over a relatively short period. These findings raise the question of how visual sensory neurons shape and keep their selectivity. Pioneering work by Simoncelli and Olshausen (2001) showed that natural image statistics shape V1-like response patterns in artificial neural networks. Furthermore, electrophysiological studies have captured an unsupervised change of representation in IT neurons in a manner dependent on the visual experience (Li and DiCarlo, 2010; Jia et al., 2021). Together with these results, we consider that the visual system may have the ability to shape its own activity depending on its input statistics, even in the short term. If this is the case, active sampling of what to see (here, the fixations and saccades) and their sequence is an important factor in visual object representation. Many previous experiments on visual neuronal activity assumed that neuronal selectivity or representation do not change much across stimuli presentation. However, this perspective might limit a comprehensive understanding of the visual system. Our report suggests that the conventional approach to visual information processing, which has been primarily based on studies conducted under passive conditions, needs to be revised. Although this study did not reach the point of constructing an object coding model under active conditions, constructing such an encoding model under active conditions is an important future direction.

### Relationship to previous studies on adaptation

We observed a reduction in firing rates in all the recorded areas in later fixations. Previous studies have recognized the firing rate reduction in response to repeatedly presented stimuli as adaptation or repetition suppression (RS). Adaptation or RS has been reported to show diverse durations, has been observed in various visual areas, and has been suggested to have multiple different underlying neuronal mechanisms (Wiggs and Martin, 1998; Patterson et al., 2013; Solomon and Kohn, 2014). However, RS has mostly been examined under passive conditions. From studies of fixation tasks, the strength of RS is known to depend on the interval between presentations of the same stimulus, stimulus duration, and on the similarity between the recently viewed object and the subsequent object (“cross-adaptation”). In our study, these factors are not controlled by the experimenter, but the subject (animal) controls the order of fixations in free viewing. Thus, exact comparisons of the RS effect between free viewing and fixation tasks are not simple. Having understood these differences, when we intentionally compared our observations with free viewing tasks with those of previous reports, we found similarities. The proportion of neurons that showed suppression in our study in IT (20–30%) was similar to the values initially reported for fixation or delayed-matching-to-sample tasks (>1/3 of neurons from Miller et al., 1991; and ∼1/4 of those in Ringo, 1996). The timing and duration of suppression in our study, which was 100–200 ms after fixation onset (FODR2), overlaps with the previously reported 160- to 200-ms range (Liu et al., 2009; Vogels, 2016). These reports regarding RS are also consistent with the results of our fixation task experiments. Thus, the firing rate reduction in our free viewing task is indistinguishable from the phenomenon called “RS” in the previous reports regarding the percentage of neurons and the timing and duration of suppression. However, the effect size was relatively small, which is possibly explained by the fact that the particular order of fixation that the animals took was never the same as the order and duration of stimulus presentation in the controlled fixation tasks. The early start of suppression in our study (FODR1) may reflect a process specific to active exploration, such as the predictability of the input image, which was missing in previous reports that employed the passive viewing paradigm (such as Liu et al., 2009; Vogels, 2016). In addition to these active-passive differences, the interstimulus interval was longer than the saccade duration (<50 ms) in previously reported fixation tasks and our fixation tasks (200 ms). It is possible that these timing differences also affected the timing of suppression.

For the lower visual areas (V1 and V2), it is difficult to compare our observations with many previous reports because we used more intricate objects as stimuli. Most reports on V1 adaptation have used simple, parameterized stimuli, such as grating patterns, and strictly controlled the retinal position of stimulus presentation. However, Peter et al. (2021) used object stimuli and found a reduction in V1 MUAs with repeated presentations in a passive condition. They reported that the reduction started 33–57 ms after stimulus onset, which corresponds to the FODR1 period in our study. This result contradicts our findings in the fixation task. This discrepancy may be because of differences in the stimuli presentation order. They showed the same stimuli consecutively. Interestingly, in our study, a considerable fraction of V1 and V2 neurons reduced their firing rates depending on the behavioral context, despite their retinal images not being strictly the same. This observation shows that behavioral contexts, such as experience, which we evaluated by the fixation number in the free viewing trials, can affect neural activity and override “instantaneous visual inputs,” even in the lower visual areas.

Recently, Williams and Olson (2022) reported that RS occurred in V2 using complex object images in the fixation task. This observation was consistent with the results of our free viewing task. In addition, they reported the possibility of a difference in the mechanism of RS between V2 and IT using complex object images. They demonstrated that RS occurred when the same stimulus was shown in different retinal positions in IT but not in V2. Conversely, different object stimuli caused RS in V2 but not in IT. Because the tuning of V2 is not primarily based on objects but on lower-level image features, the observation that the different objects evoked RS is understandable. Additionally, considering the position invariance of IT, it is also reasonable that RS occurs with stimuli presented in different locations. However, taking these differences into account, it indicates that RS in the higher visual area cannot be explained simply by a scheme where the suppression in lower visual areas directly causes it to occur in a higher visual area. A comparison study between different areas using the same condition would be important in this regard.

### Meaning of firing rate reduction in object coding

The mechanism of adaptation, and its benefits for visual processing, are still under debate. One hypothesis is the sharpening model, which assumes that adaptation causes sharpened tuning of individual neurons (Desimone, 1996; Wiggs and Martin, 1998; Müller et al., 1999). This hypothesis has been extensively studied in IT and postulates that the activity of neurons that are comparatively less optimal to the adapter (a stimulus that is used for repeated presentation) could be reduced. In this way, repeated presentation of an adapter prunes unnecessary neuronal activity and accentuates the response of neurons coding the adapter stimulus. However, this hypothesis has been challenged by electrophysiological reports showing that RS does not selectively sharpen neural representation by demonstrating that the shape tuning curve does not sharpen with RS (De Baene and Vogels, 2010). Furthermore, no correlation was observed by Sawamura et al. (2006) and McMahon and Olson (2007) between the strength of adaptation and the firing rate. In these studies, adaptation and selectivity were measured for individual neurons, but population activity was not considered. Kaliukhovich et al. (2013) examined single-unit activities and simultaneously recorded MUAs during the repeated presentation of object stimuli. They observed both enhancement and diminishment of object discriminability by single neurons, depending on their tuning (effectiveness for neural response) to the objects. In other words, response reduction because of adaptation tended to be greater for preferred objects, resulting in increased discriminability when the adaptor is a less responsive object. Additionally, they demonstrated that the discrimination accuracy of object identity, as measured by simultaneously recorded MUAs, is lower under repeated stimuli. This latter finding does not contradict our result that repeated fixation on an object slightly decreases discrimination accuracy measured by LDA. There are some difficulties and limitations to evaluating population activity under a lower firing rate, and we discuss them below.

It has been suggested that the situation is not as simple as only reductions in the firing rate and tuning sharpening, even in V1 (Wissig and Kohn, 2012; Patterson et al., 2013; Solomon and Kohn, 2014). The sharpening model cannot simply be extended to population coding; the effect will be more complicated and will depend on the relationship between the underlying tuning of individual neurons to the adapter and noise correlation between neurons. This interaction affects the amount of information the population can convey (Gutnisky and Dragoi, 2008; Cortes et al., 2012; Shamir, 2014; Kohn et al., 2016). In partial support of the sharpening model, we observed increases in the sparseness of individual neurons in later fixations or a later part of single fixations. However, the sharpening hypothesis assumes that individual neurons have their own optimal stimuli that evoke the strongest activity and assumes that the reduction of the response to nonoptimal stimuli sharpens relative activity to the optimal stimuli. We hypothesize that changes in firing rate depend solely on experience and that the cell’s tuning is not permanent.

It has been hypothesized that adaptation sharpens firing timing. Previous reports have shown that the γ frequency spike synchronization increases after adaptation in V1 and V4 (Hansen and Dragoi, 2011; Wang et al., 2011; Brunet et al., 2014; and more recently, Peter et al., 2021). Kaliukhovich and Vogels (2012) reported similar phenomena in IT, with RS changing the coherence between LFPs in different laminar structures and between MUA and LFPs. However, the frequency range differs between reports, and increases and decreases in the magnitude of synchronization have been observed. Accumulation of more evidence is necessary to evaluate the validity of this hypothesis. We did not examine coherence or synchronization in this study. Whether such synchronous activity changes during repeated fixations on the same object is a pertinent question with regard to the potential functional advantage of this phenomenon.

A further hypothesis is that RS is explained by the predictive coding framework (Summerfield et al., 2008; Auksztulewicz and Friston, 2016). Top-down predictions serve to inhibit or suppress bottom-up sensory evidence, with residual activity in the lower levels of the cortical hierarchy serving as “prediction error” signals that are, in turn, relayed to the higher levels. To verify this hypothesis, it is essential to identify the “prediction error” and “prediction signals” separately. For this purpose, layer-specific recordings of the lower and higher areas are required. Unfortunately, we were not able to separate IT neural activity in a layer-specific manner. The task paradigm is also not optimal for examining this hypothesis. Nevertheless, our observations do not contradict this hypothesis.

The above hypotheses emphasize the interpretation of adaptation as an active function necessary for information processing in the cortex. Our results show that repeated fixations affect neural activity in active vision and further supports the view that this phenomenon is a natural and necessary process for visual recognition.

### Latency variation of IT neurons

The latency of IT responses is generally 80–100 ms. There was a slight shift of the peak to an earlier time in the averaged firing rate in later fixations (Fig. 2*B*). This observation is potentially interesting. We found that some recorded neurons had relatively early latencies (<60 ms). Additionally, there were other factors, such as the influence of the previous fixation (Sheinberg and Logothetis, 2001) or predictive activity. We did not pursue this further in this study as we needed more samples to identify it. Investigating the details of this early response, including whether the response latency decreases, is something we would like to consider in a future study.

### Response firing rate of IT neurons

The firing rate of our IT neurons is lower than that reported in some previous studies (Lehky et al., 2008; Kaliukhovich and Vogels, 2016). Several possible reasons account for this disparity, including the following. It has been consistently demonstrated that the responses of IT neurons decrease when a second stimulus is introduced into the background (Sato, 1989; Rolls and Tovee, 1995b; Missal et al., 1999; Zoccolan et al., 2005, 2007; De Baene et al., 2007) or in cluttered conditions during free viewing (Sheinberg and Logothetis, 2001), as opposed to when a stimulus is presented in isolation during a fixation task. Consequently, the response is anticipated to be sparser in comparison to the response elicited by isolated stimuli in the fixation task.

### Limitations of linear discriminant analysis

The decrease in cosine similarity and the increase in sparseness naturally predict the increase in object discrimination accuracy in LDA. However, the results showed no increase in discrimination accuracy. There are several possible explanations for this. First, cosine similarity is a measure that does not consider the vector’s magnitude. For example, if the firing rate for individual neural activity is 2-fold, it can have the same cosine similarity. In contrast, LDA can use the difference in the magnitude of the vector as information to discriminate. As the firing rate is generally higher in first fixations, the objects of first fixations are easier to discriminate. Second, not all pairs of cosine similarities decreased across the fixation order, which means that the differences in cosine similarities varies, and the effect was not so large, as a whole, to make LDA discrimination easier in later fixations. Third, the number of simultaneously recorded neurons was so small that we might not have seen the whole picture. The current dataset of the pooled samples, across mixes 2–3 and 4–6, might have taken away the effect observed in the cosine similarity analysis and the sparseness analysis. All the above might have contributed to the results. Although we did not observe an increase in LDA accuracy, we showed slight or no decrease across fixation orders. These results still support the hypothesis that dynamic change in object representation lets a smaller number of spikes allow for discrimination of individual objects in a scene in free viewing.

### Eye movement behavior

Behaviorally, primates, including humans and macaque monkeys, employ similar strategies in visual exploration in free viewing. Two distinct scan modes, global and local, appear during scene viewing (Pannasch et al., 2008; Marsman et al., 2013; Ito et al., 2017). In global scan mode, multiple objects scattered in the scene are fixated on, one after another, with short fixation durations. In local scan mode, the fixation duration is longer, and the same object is repeatedly fixated on. We hypothesized that this change in the scan mode affected neuronal activity, hence the neurons changed their activity across fixations depending on the fixation order. The current study demonstrated that visual neural activities differ in accordance with behavioral dynamics between early fixations and repeated later fixations on the same object. In our experiments, first fixations happened relatively early and roughly corresponded to the ambient exploration mode. Some of the later fixations, especially second+ fixations corresponded to the local scan mode. Although the observations by Ito et al. (2017) that showed switching between two modes, were not directly referenced in this study, it is highly probable that the “mode switch” correlates to the population neural activity change observed in our data.

## Synthesis

Reviewing Editor: Niko Busch, Westfalische Wilhelms-Universitat Munster Decisions are customarily a result of the Reviewing Editor and the peer reviewers coming together and discussing their recommendations until a consensus is reached. When revisions are invited, a fact-based synthesis statement explaining their decision and outlining what is needed to prepare a revision will be listed below. The following reviewer(s) agreed to reveal their identity: Rufin Vogels, Kohitij Kar.

# Synthesis

This manuscript has been reviewed by two experts. Their comments are included at the bottom of this letter. Both reviews highlight the merit of your study, but they also agree on the need to improve the manuscript further. Specifically, while the reviewers appreciate the appeal of the free viewing paradigm, they also point out its downsides in terms of controllability. I have discussed this issue with the reviewers and I agree with their concerns that claims about increased neuronal selectivity and distinctiveness with repeated fixations are not fully convincing yet, and should be substantiated with additional analyses. Moreover, both reviewers are concerned with what appears to be an unusually low firing rate especially of IT neurons, which may render these data unreliable. I encourage you to carefully address each of these points.

# Reviewer 1

This paper is a revised version of a previous submission. To obtain an unbiased opinion on the paper I did not read the rebuttal of the authors of this revision.

The large majority of studies of visual cortical processing in awake monkeys measured the responses of neurons during controlled fixation. Here, the authors measured the responses of visual cortical neurons during free viewing of objects, examining the effect of revisits of the same object during free viewing on the response strength and selectivity. This is an ill-studied but difficult question and for sure important. Another strength of the paper is that they recorded from 3 visual areas in the same monkeys: V1, V2, and IT, allowing a comparison of the responses among the areas. Overall, the (small) effects appear to fit what one would expect, i.e. repetition suppression while re-fixating an object. That said, I was not impressed by the outcome of this study as explained below.

Main comments.

1. The averaged responses, especially in IT, are rather low: at the population level, we are dealing with average net peak firing rates of less than 2 spikes/s for IT (Figure 2)! The sizes of the reported effects, in particular for the selectivity measures, are also small and not consistent between different analyses: for instance, they obtain small effects of object revisits on cosine similarity but no effect or an opposite effect when performing Linear Discriminant Analysis. The reduction of the responses with revisits is stronger but expected based on repetition suppression.

2. Related to the above point: the effects of object-revisits during free-viewing are expected to be more variable than those obtained with fixed stimulus presentation schedules and under controlled fixation. The strength of repetition suppression is known to depend on the interval between presentations of the same stimulus, which is variable during free-viewing, stimulus duration, which is likely to be variable during free-viewing, and on the similarity between the recently viewed object and the subsequently seen object (“cross-adaptation”), which will also vary during free-viewing. All these variables can impact the extent of repetition suppression and produce noisy, variable effects during free-viewing. This makes studying and interpreting history effects during free-viewing very difficult. This can explain some of the small effect sizes. This should be discussed.

3. They show pooled recording data of two monkeys. To what extent were each of the reported effects, in particular those for sparseness and cosine similarity, present in each monkey? This should be documented to see how consistent the effects are across the two subjects.

4. In IT, there appears to be an effect on the mean response of revisits before fixation onset and immediately after fixation onset (Figure 2). Is this because of the large receptive field of IT neurons or because of including very low responsive neurons, i.e. noisy data? Also, one should perform a baseline (pre- or peri-fixation) correction when comparing the responses across revisits, because the baseline (pre- and peri-fixation) differs between revisit conditions.

5. It appears that when testing the significances of the pairwise computed cosine similarities between the fixation revisits the authors treat the object pairs as independent samples. However, for sure these are not independent (similarity between a and b, a and c, and b and c are not independent). These dependencies should be taken into account when testing the significance.

6. The comparison of the free-viewing data with the fixation task is difficult to interpret and I suggest removing it from the manuscript. The reason is that the inter-stimulus intervals, number of objects, etc are very different between the two tasks, making the comparison flawed.

Minor comments.

1. The authors write that “the 2nd+ and re-visit fixations had a commonality in terms of behavioral context: both fixations occurred when the monkey was already fixated on a previously fixated object.” However, there is a fundamental difference between these two conditions since the 2nd+ fixations stay within the same object, which is expected to increase repetition suppression. This should be clarified.

2. The authors should indicate significant effects (preferably with exact p values) in all the Figures. Also, they should explain the bar plots (medians are plotted and boxes are quartiles, right?).

3. The authors should do a better job of relating their findings to previous studies, especially those on repetition suppression. For instance, Williams and Olson (J. Neurophys., 2022) showed repetition suppression in V2 and compared that with repetition suppression in IT. This was done under fixation conditions. Similarly, Kaliukhovich et al (J. Cogn Neurosci, 2013) examined the effects of adaptation on object classification, similar to what the authors did here with the LDA. Their findings, obtained during controlled fixation, should be related to the present (much weaker) effects obtained during free-viewing.

4. The manuscript contains several sentences that are grammatically incorrect. Please have the manuscript read by a native English speaker.

# Reviewer 2

In this article, the authors perform exploratory analyses to characterize the “relationship between spiking activity [of V1, V2, and IT] and the order of fixations and the fixated objects [during active vision].”

In general, this is in an important topic of research. Most attempts to test encoding models of the primate ventral stream are done during passive fixation trials. The variations of neural activity during active vision are often overlooked and it is important to study these. The authors say they want “To investigate neural representation of objects in this [active vision] process”. One reasonable objective therefore could be that the authors should identify an improved encoding model of the neurons that responds to visual stimuli during active vision. However, here the authors do not provide an image-computable model as their inference but rather provide an explorative characterization of the neural activity. That is still valuable but I would recommend certain controls that will enable ruling out the confounds that limit the inferences. .

The authors should discuss this in the Discussion section as a limitation of the study and that the responses should be used in the future to help build better encoding models.

Major concerns:

How reliable are recordings across the three areas?Are recordings in V1 and V2 more reliable than recordings in IT? That would reduce the likelihood of finding more dynamically distinct activity regions in the responses of IT neurons - which would support the results reported. Can I author rule this out?

For Figure 3, one needs to estimate whether the object ID determination on fixation 1 is reliable. Sorting firing rates per object will always result in a figure like 3A (left panel). If this is not a reliable pattern, then even when the monkey fixates on these objects on a different trial for the first time, the results will look like Fig. 3A (middle panel). So to make sense of the middle and right panel, the variance in the ranking of the left panel is important. Errorbars are necessary.

Lower response = lower signal (more noise), and therefore correlating with this lower signal will yield lower values (i.e. dissimilarity between population patterns). This confound must be addressed to adequately support the claims of the paper.

Minor:

1. The text of the paper needs to be revised. For instance, e.g. line 369 “showed a slight decrease”. Quantify the change.

2. Sometimes figures are Fig. Sometimes they are Figure.
