# Supplementary material for: Neuronal Population Activity in Macaque Visual Cortices Dynamically Changes through Repeated Fixations in Active Free Viewing
Source: eNeuro. 2023 Oct 18;10(10):ENEURO.0086-23.2023. doi: 10.1523/ENEURO.0086-23.2023 (PMC10591287; doi:10.1523/ENEURO.0086-23.2023)
Supplement: Extended Data Table 1-1 — Comparison of saccade amplitude. The p-values were determined by the rank-sum test (two sided). The effect size is the Cliff’s δ effect size. Download Table 1-1, DOCX file. [file enu-eN-NWR-0086-23-s05.docx]

| **categories compared** | **n** | **mean1** | **mean2** | **p value**  **(Mann Whitney -U)** | **p < 0.05** | **p < 0.01** | **effect size** |
| --- | --- | --- | --- | --- | --- | --- | --- |
| **1st vs 2nd+** | 91474 | 8.8958 | 0.8910 | 0 |  | * | 0.9423 |
| **1st vs rev** | 65730 | 8.8958 | 7.5888 | 0 |  | * | 0.1511 |
| **2nd+ vs rev** | 45289 | 0.8910 | 7.5888 | 0 |  | * | 0.9012 |
