# Supplementary material for: Neuronal Population Activity in Macaque Visual Cortices Dynamically Changes through Repeated Fixations in Active Free Viewing
Source: eNeuro. 2023 Oct 18;10(10):ENEURO.0086-23.2023. doi: 10.1523/ENEURO.0086-23.2023 (PMC10591287; doi:10.1523/ENEURO.0086-23.2023)
Supplement: Extended Data Table 1-2 — Comparison of fixation position overlap. The p-values were determined by the signed-rank test (two sided). The effect size is the Cliff’s δ effect size. Download Table 1-2, DOCX file. [file enu-eN-NWR-0086-23-s06.docx]

| **categories compared** | **n** | **mean1** | **mean2** | **p value**  **(signed-rank)** | **p < 0.05** | **p < 0.01** | **effect size** |
| --- | --- | --- | --- | --- | --- | --- | --- |
| **(1st-2nd+) vs (1st-rev)** | 1176 | 0.7523 | 0.8839 | 5.881x10-182 |  | * | 0.8252 |
| **(1st-2nd+) vs 2nd+-rev)** | 1176 | 0.7523 | 0.7550 | 0.2883 |  |  | 0.0211 |
| **(1st-rev) vs 2nd+-rev)** | 1176 | 0.8839 | 0.7550 | 5.994x10-184 |  | * | 0.8378 |
