# Supplementary material for: Neuronal Population Activity in Macaque Visual Cortices Dynamically Changes through Repeated Fixations in Active Free Viewing
Source: eNeuro. 2023 Oct 18;10(10):ENEURO.0086-23.2023. doi: 10.1523/ENEURO.0086-23.2023 (PMC10591287; doi:10.1523/ENEURO.0086-23.2023)
Supplement: Extended Data Table 3-1 — Sparseness comparison across fixation order. The p-values were determined by the signed-rank test (two sided). The effect size is the Cliff’s δ effect size. Download Table 3-1, DOCX file. [file enu-eN-NWR-0086-23-s07.docx]

| **area and period** | **categories compared** | **n** | **mean1** | **mean2** | **p value**  **(signed-rank)** | **p < 0.05** | **p < 0.01** | **effect size** |
| --- | --- | --- | --- | --- | --- | --- | --- | --- |
|  | **1st vs 2nd+** | 85 | 0.3090 | 0.3830 | 2.377x10-5 |  | * | 0.1961 |
| **V1 FODR1** | **1st vs re-visit** | 85 | 0.3090 | 0.4252 | 1.539x10-4 |  | * | 0.2913 |
|  | **2nd+ vs re-visit** | 85 | 0.3830 | 0.4252 | 2.410x10-3 |  | * | 0.0987 |
|  | **1st vs 2nd+** | 85 | 0.3586 | 0.4437 | 1.452x10-6 |  | * | 0.2111 |
| **V1 FODR2** | **1st vs re-visit** | 85 | 0.3586 | 0.4736 | 1.410x10-8 |  | * | 0.2725 |
|  | **2nd+ vs re-visit** | 85 | 0.4437 | 0.4736 | 0.05413 |  |  | 0.0688 |
|  | **1st vs 2nd+** | 95 | 0.3084 | 0.4247 | 2.139x10-10 |  | * | 0.3374 |
| **V2 FODR1** | **1st vs re-visit** | 95 | 0.3084 | 0.4361 | 6.847x10-13 |  | * | 0.3416 |
|  | **2nd+ vs re-visit** | 95 | 0.4247 | 0.4361 | 0.5601 |  |  | 0.0154 |
|  | **1st vs 2nd+** | 95 | 0.3359 | 0.4872 | 2.456x10-13 |  | * | 0.4013 |
| **V2 FODR2** | **1st vs re-visit** | 95 | 0.3359 | 0.4981 | 4.114x10-14 |  | * | 0.4018 |
|  | **2nd+ vs re-visit** | 95 | 0.4872 | 0.4981 | 0.5136 |  |  | 0.0153 |
|  | **1st vs 2nd+** | 318 | 0.4786 | 0.5383 | 5.509x10-10 |  | * | 0.1435 |
| **IT FODR1** | **1st vs re-visit** | 318 | 0.4786 | 0.5719 | 5.849x10-24 |  | * | 0.2150 |
|  | **2nd+ vs re-visit** | 318 | 0.5383 | 0.5719 | 9.665x10-5 |  | * | 0.0842 |
|  | **1st vs 2nd+** | 318 | 0.4753 | 0.5650 | 1.084x10-13 |  | * | 0.2060 |
| **IT FODR2** | **1st vs re-visit** | 318 | 0.4753 | 0.5874 | 2.322x10-31 |  | * | 0.2525 |
|  | **2nd+ vs re-visit** | 318 | 0.5650 | 0.5874 | 0.006410 |  | * | 0.0512 |
