# Supplementary material for: Neuronal Population Activity in Macaque Visual Cortices Dynamically Changes through Repeated Fixations in Active Free Viewing
Source: eNeuro. 2023 Oct 18;10(10):ENEURO.0086-23.2023. doi: 10.1523/ENEURO.0086-23.2023 (PMC10591287; doi:10.1523/ENEURO.0086-23.2023)
Supplement: Extended Data Table 3-2 — Sparseness comparison between FODR1 and FODR2. The p-values were determined by the signed-rank test (two sided). The effect size is the Cliff’s δ effect size. Download Table 3-2, DOCX file. [file enu-eN-NWR-0086-23-s08.docx]

| **area** | **fixation** | **categories compared** | **n** | **mean1** | **mean2** | **p value**  **(signed-rank)** | **p < 0.05** | **p < 0.01** | **effect size** |
| --- | --- | --- | --- | --- | --- | --- | --- | --- | --- |
|  | **1st** | **FODR1 vs FODR2** | 85 | 0.3090 | 0.3586 | 0.01111 | * |  | 0.0998 |
| **V1** | **2nd+** | **FODR1 vs FODR2** | 85 | 0.3830 | 0.4437 | 0.0002979 |  | * | 0.1405 |
|  | **re-visit** | **FODR1 vs FODR2** | 85 | 0.4252 | 0.4736 | 0.003257 |  | * | 0.1147 |
|  | **1st** | **FODR1 vs FODR2** | 95 | 0.3084 | 0.3359 | 0.2151 |  |  | 0.0668 |
| **V2** | **2nd+** | **FODR1 vs FODR2** | 95 | 0.4247 | 0.4872 | 0.001646 |  | * | 0.1819 |
|  | **re-visit** | **FODR1 vs FODR2** | 95 | 0.4361 | 0.4981 | 0.003452 |  | * | 0.1527 |
|  | **1st** | **FODR1 vs FODR2** | 318 | 0.4786 | 0.4753 | 0.4232 |  |  | 0.0052 |
| **IT** | **2nd+** | **FODR1 vs FODR2** | 318 | 0.5383 | 0.5650 | 0.002186 |  | * | 0.0737 |
|  | **re-visit** | **FODR1 vs FODR2** | 318 | 0.5719 | 0.5874 | 0.03863 | * |  | 0.0383 |
