# Supplementary material for: Neuronal Population Activity in Macaque Visual Cortices Dynamically Changes through Repeated Fixations in Active Free Viewing
Source: eNeuro. 2023 Oct 18;10(10):ENEURO.0086-23.2023. doi: 10.1523/ENEURO.0086-23.2023 (PMC10591287; doi:10.1523/ENEURO.0086-23.2023)
Supplement: Extended Data Table 4-1 — Comparison of cosine similarities across fixation orders. The p-values were determined by the Kolmogorov–Smirnov test (two sided). The effect size is the Cliff’s δ effect size. Download Table 4-1, DOCX file. [file enu-eN-NWR-0086-23-s09.docx]

| **area and period** | **categories compared** | **n** | **mean1** | **mean 2** | **p value**  **(Kolmogorov-Smirnov)** | **p < 0.05** | **p < 0.01** | **effect size** |
| --- | --- | --- | --- | --- | --- | --- | --- | --- |
|  | **1st vs 2nd+** | 991 | 0.6422 | 0.5920 | 1.2629x10-14 |  | * | 0.05017 |
| **V1 FODR1** | **1st vs re-visit** | 991 | 0.6422 | 0.6023 | 2.1762x10-6 |  | * | 0.03987 |
|  | **2nd+ vs re-visit** | 991 | 0.5920 | 0.6023 | 0.01347 | * |  | 0.01030 |
|  | **1st vs 2nd+** | 991 | 0.5971 | 0.5112 | 6.3643x10-16 |  | * | 0.08591 |
| **V1 FODR2** | **1st vs re-visit** | 991 | 0.5971 | 0.5180 | 2.8817x10-5 |  | * | 0.07906 |
|  | **2nd+ vs re-visit** | 991 | 0.5112 | 0.5180 | 0.08151 |  |  | 0.006843 |
|  | **1st vs 2nd+** | 1210 | 0.4669 | 0.4223 | 3.5809x10-12 |  | * | 0.04460 |
| **V2 FODR1** | **1st vs re-visit** | 1210 | 0.4669 | 0.4417 | 0.8402x10-9 |  | * | 0.02524 |
|  | **2nd+ vs re-visit** | 1210 | 0.4223 | 0.4417 | 0.02974 |  |  | 0.01936 |
|  | **1st vs 2nd+** | 1210 | 0.4144 | 0.3332 | 1.0008x10-54 |  | * | 0.08118 |
| **V2 FODR2** | **1st vs re-visit** | 1210 | 0.4144 | 0.3854 | 6.288x10-15 |  | * | 0.02891 |
|  | **2nd+ vs re-visit** | 1210 | 0.3332 | 0.3854 | 5.8287x10-16 |  |  | 0.05227 |
|  | **1st vs 2nd+** | 2342 | 0.3804 | 0.3127 | 6.2662x10-51 |  | * | 0.06770 |
| **IT FODR1** | **1st vs re-visit** | 2342 | 0.3804 | 0.3268 | 1.2212x10-34 |  | * | 0.05364 |
|  | **2nd+ vs re-visit** | 2342 | 0.3127 | 0.3268 | 2.6864x10-3 |  | * | 0.01406 |
|  | **1st vs 2nd+** | 2342 | 0.3630 | 0.2822 | 2.2737x10-78 |  | * | 0.08075 |
| **IT FODR2** | **1st vs re-visit** | 2342 | 0.3630 | 0.2953 | 1.0989x10-53 |  | * | 0.06767 |
|  | **2nd+ vs re-visit** | 2342 | 0.2822 | 0.2953 | 0.002166 |  | * | 0.01306 |
