# Supplementary material for: Neuronal Population Activity in Macaque Visual Cortices Dynamically Changes through Repeated Fixations in Active Free Viewing
Source: eNeuro. 2023 Oct 18;10(10):ENEURO.0086-23.2023. doi: 10.1523/ENEURO.0086-23.2023 (PMC10591287; doi:10.1523/ENEURO.0086-23.2023)
Supplement: Extended Data Table 4-2 — Comparison of cosine similarities between FODR1 and FODR2. The p-values were determined by the Kolmogorov–Smirnov test (two sided). The effect size is the Cliff’s δ effect size. Download Table 4-2, DOCX file. [file enu-eN-NWR-0086-23-s10.docx]

| **area** | **fixation** | **categories compared** | **n** | **mean1** | **mean2** | **p value**  **(Kolmogorov-Smirnov)** | **p < 0.05** | **p < 0.01** | **effect size** |
| --- | --- | --- | --- | --- | --- | --- | --- | --- | --- |
|  | **1st** | **FODR1 vs FODR2** | 991 | 0.6422 | 0.5971 | 3.360x10-12 |  | * | 0.04508 |
| **V1** | **2nd+** | **FODR1 vs FODR2** | 991 | 0.5920 | 0.5112 | 1.013x10-20 |  | * | 0.08081 |
|  | **re-visit** | **FODR1 vs FODR2** | 991 | 0.6023 | 0.5181 | 0.7336x10-23 |  | * | 0.08427 |
|  | **1st** | **FODR1 vs FODR2** | 1210 | 0.4669 | 0.4143 | 6.0663x10-8 |  | * | 0.05259 |
| **V2** | **2nd+** | **FODR1 vs FODR2** | 1210 | 0.4223 | 0.3332 | 8.1268x10-24 |  | * | 0.08917 |
|  | **re-visit** | **FODR1 vs FODR2** | 1210 | 0.4417 | 0.3854 | 9.7794x10-8 |  | * | 0.05626 |
|  | **1st** | **FODR1 vs FODR2** | 2342 | 0.3801 | 0.3630 | 9.9349x10-4 |  | * | 0.01749 |
| **IT** | **2nd+** | **FODR1 vs FODR2** | 2342 | 0.3127 | 0.2822 | 9.6910x10-11 |  | * | 0.03053 |
|  | **re-visit** | **FODR1 vs FODR2** | 2342 | 0.3268 | 0.2953 | 4.2421x10-12 |  | * | 0.03152 |
