# Supplementary material for: Neuronal Population Activity in Macaque Visual Cortices Dynamically Changes through Repeated Fixations in Active Free Viewing
Source: eNeuro. 2023 Oct 18;10(10):ENEURO.0086-23.2023. doi: 10.1523/ENEURO.0086-23.2023 (PMC10591287; doi:10.1523/ENEURO.0086-23.2023)
Supplement: Extended Data Table 5-1 — Comparison of cosine similarities between second1 and second2 fixations. p-values were determined by the s Kolmogorov–Smirnov test (two sided). The effect size is the Cliff’s δ effect size. Download Table 5-1, DOCX file. [file enu-eN-NWR-0086-23-s11.docx]

| **area** | **period** | **categories compared** | **n** | **mean1** | **mean2** | **p value**  **(Kolmogorov-Smirnov)** | **p < 0.05** | **p < 0.01** | **effect size** |
| --- | --- | --- | --- | --- | --- | --- | --- | --- | --- |
| **V1** | **FODR1** | 2nd1 vs 2nd2 | 788 | 0.8653 | 0.7905 | 1.483x10-16 |  | * | 0.2554 |
|  | **FODR2** | 2nd1 vs 2nd2 | 788 | 0.8291 | 0.7144 | 1.197x10-21 |  | * | 0.2880 |
| **V2** | **FODR1** | 2nd1 vs 2nd2 | 910 | 0.7784 | 0.6970 | 6.869x10-10 |  | * | 0.1626 |
|  | **FODR2** | 2nd1 vs 2nd2 | 910 | 0.7381 | 0.5640 | 1.204x10-39 |  | * | 0.2880 |
| **IT** | **FODR1** | 2nd1 vs 2nd2 | 1832 | 0.7462 | 0.6042 | 2.496x10-47 |  | * | 0.3164 |
|  | **FODR2** | 2nd1 vs 2nd2 | 1832 | 0.7431 | 0.6008 | 1.013x10-55 |  | * | 0.3149 |
