# Supplementary material for: Neuronal Population Activity in Macaque Visual Cortices Dynamically Changes through Repeated Fixations in Active Free Viewing
Source: eNeuro. 2023 Oct 18;10(10):ENEURO.0086-23.2023. doi: 10.1523/ENEURO.0086-23.2023 (PMC10591287; doi:10.1523/ENEURO.0086-23.2023)
Supplement: Extended Data Table 5-2 — Comparison of cosine similarities between re-visit1 and re-visit2 fixations. p-values were determined by the Kolmogorov–Smirnov test (two sided). The effect size is the Cliff’s δ effect size. (h) indicates that mean2 is higher than mean1. Download Table 5-2, DOCX file. [file enu-eN-NWR-0086-23-s12.docx]

| **area** | **period** | **categories compared** | **n** | **mean1** | **mean2** | **p value**  **(Kolmogorov-Smirnov)** | **p < 0.05** | **p < 0.01** | **effect size** |
| --- | --- | --- | --- | --- | --- | --- | --- | --- | --- |
| **V1** | **FODR1** | re-visit1 vs re-visit2 | 534 | 0.7877 | 0.8083 | 0.1108 |  |  | 0.0537 |
|  | **FODR2** | re-visit1 vs re-visit2 | 534 | 0.7260 | 0.7506 | 0.1945 |  |  | 0.0431 |
| **V2** | **FODR1** | re-visit1 vs re-visit2 | 620 | 0.6849 | 0.6702 | 0.7798 |  |  | 0.0198 |
|  | **FODR2** | re-visit1 vs re-visit2 | 620 | 0.6480 | 0.6495 | 0.9991 |  |  | 0.0431 |
| **IT** | **FODR1** | re-visit1 vs re-visit2 | 1654 | 0.5620 | 0.5795 | 0.006444 |  | *(h) | 0.0367 |
|  | **FODR2** | re-visit1 vs re-visit2 | 1654 | 0.5703 | 0.5874 | 0.01147 | *(h) |  | 0.0363 |
