# Supplementary material for: Neuronal Population Activity in Macaque Visual Cortices Dynamically Changes through Repeated Fixations in Active Free Viewing
Source: eNeuro. 2023 Oct 18;10(10):ENEURO.0086-23.2023. doi: 10.1523/ENEURO.0086-23.2023 (PMC10591287; doi:10.1523/ENEURO.0086-23.2023)
Supplement: Extended Data Table 5-3 — Comparison of cosine similarities across saccade order, first, and mix second to fifth fixations. p-values were determined by the Kolmogorov–Smirnov test (two sided). The effect size is the Cliff’s δ effect size. Download Table 5-3, DOCX file. [file enu-eN-NWR-0086-23-s13.docx]

| **area and period** | **categories compared** | **n** | **mean1** | **mean2** | **p value**  **(Kolmogorov-Smirnov )** | **p < 0.05** | **p < 0.01** | **effect size** |
| --- | --- | --- | --- | --- | --- | --- | --- | --- |
|  | **1st vs mix2** | 846 | 0.6380 | 0.5176 | 5.730x10-54 |  | * | 0.4329 |
| **V1 FODR1** | **1st vs mix3** | 846 | 0.6380 | 0.5322 | 3.650x10-41 |  | * | 0.3794 |
|  | **1st vs mix4** | 846 | 0.6380 | 0.5105 | 2.650x10-53 |  | * | 0.4414 |
|  | **1st vs mix5** | 846 | 0.6380 | 0.5363 | 7.166x10-32 |  | * | 0.3217 |
|  | **1st vs mix2** | 846 | 0.58841 | 0.4871 | 5.762x10-27 |  | * | 0.3246 |
| **V1 FODR2** | **1st vs mix3** | 846 | 0.58841 | 0.4983 | 1.364x10-20 |  | * | 0.2734 |
|  | **1st vs mix4** | 846 | 0.58841 | 0.4672 | 5.762x10-27 |  | * | 0.3365 |
|  | **1st vs mix5** | 846 | 0.58841 | 0.4770 | 4.011x10-23 |  | * | 0.3078 |
|  | **1st vs mix2** | 989 | 0.46866 | 0.3925 | 1.579x10-29 |  | * | 0.3027 |
| **V2 FODR1** | **1st vs mix3** | 989 | 0.46866 | 0.3928 | 2.243x10-31 |  | * | 0.2633 |
|  | **1st vs mix4** | 989 | 0.46866 | 0.3834 | 3.397x10-41 |  | * | 0.2840 |
|  | **1st vs mix5** | 989 | 0.46866 | 0.3887 | 2.243x10-31 |  | * | 0.2347 |
|  | **1st vs mix2** | 989 | 0.41290 | 0.3529 | 4.479x10-29 |  | * | 0.2870 |
| **V2 FODR2** | **1st vs mix3** | 989 | 0.41290 | 0.3154 | 2.574x10-68 |  | * | 0.4465 |
|  | **1st vs mix4** | 989 | 0.41290 | 0.2960 | 2.828x10-77 |  | * | 0.4887 |
|  | **1st vs mix5** | 989 | 0.41290 | 0.2844 | 2.309x10-88 |  | * | 0.4942 |
|  | **1st vs mix2** | 1835 | 0.39725 | 0.3495 | 1.293x10-21 |  | * | 0.2163 |
| **IT FODR1** | **1st vs mix3** | 1835 | 0.39725 | 0.3268 | 2.271x10-39 |  | * | 0.2966 |
|  | **1st vs mix4** | 1835 | 0.39725 | 0.3186 | 2.071x10-46 |  | * | 0.3166 |
|  | **1st vs mix5** | 1835 | 0.39725 | 0.3025 | 1.608x10-70 |  | * | 0.3501 |
|  | **1st vs mix2** | 1835 | 0.38913 | 0.3400 | 3.459x10-22 |  | * | 0.2123 |
| **IT FODR1** | **1st vs mix3** | 1835 | 0.38913 | 0.3117 | 1.023x10-42 |  | * | 0.3188 |
|  | **1st vs mix4** | 1835 | 0.38913 | 0.2802 | 1.815x10-85 |  | * | 0.4305 |
|  | **1st vs mix5** | 1835 | 0.38913 | 0.3024 | 1.617x10-61 |  | * | 0.3366 |
