# Supplementary material for: Neuronal Population Activity in Macaque Visual Cortices Dynamically Changes through Repeated Fixations in Active Free Viewing
Source: eNeuro. 2023 Oct 18;10(10):ENEURO.0086-23.2023. doi: 10.1523/ENEURO.0086-23.2023 (PMC10591287; doi:10.1523/ENEURO.0086-23.2023)
Supplement: Extended Data Table 5-4 — Slopes of the mean cosine similarities by linear least-square fitting. Download Table 5-4, DOCX file. [file enu-eN-NWR-0086-23-s14.docx]

| **area** | **time** | **slope** |
| --- | --- | --- |
| **V1** | **FODR1** | -0.021057 |
|  | **FODR2** | -0.02427 |
| **V2** | **FODR1** | -0.016895 |
|  | **FODR2** | -0.031383 |
| **IT** | **FODR1** | -0.022038 |
|  | **FODR2** | -0.23314 |
