# Supplementary material for: Neuronal Population Activity in Macaque Visual Cortices Dynamically Changes through Repeated Fixations in Active Free Viewing
Source: eNeuro. 2023 Oct 18;10(10):ENEURO.0086-23.2023. doi: 10.1523/ENEURO.0086-23.2023 (PMC10591287; doi:10.1523/ENEURO.0086-23.2023)
Supplement: Extended Data Table 5-5 — Comparison of cosine similarities between FODR1 and FODR2. The p-values were determined by the Kolmogorov–Smirnov test (two sided). The effect size is the Cliff’s δ effect size. Download Table 5-5, DOCX file. [file enu-eN-NWR-0086-23-s15.docx]

| **area** | **fixations** | **categories compared** | **n** | **mean1** | **mean2** | **p value**  **(Kolmogorov-Smirnov)** | **p < 0.05** | **p < 0.01** | **effect size** |
| --- | --- | --- | --- | --- | --- | --- | --- | --- | --- |
|  | **1st** | **FODR1 vs FODR2** | 846 | 0.6380 | 0.58841 | 9.967x10-4 |  | * | 0.1964 |
|  | **mix2** | **FODR1 vs FODR2** | 846 | 0.5176 | 0.4871 | 5.124x10-5 |  | * | 0.1151 |
| **V1** | **mix3** | **FODR1 vs FODR2** | 846 | 0.5322 | 0.4983 | 2.044x10-5 |  | * | 0.1133 |
|  | **mix4** | **FODR1 vs FODR2** | 846 | 0.5105 | 0.4672 | 4.795x10-6 |  | * | 0.1244 |
|  | **mix5** | **FODR1 vs FODR2** | 846 | 0.5363 | 0.4770 | 2.542x10-10 |  | * | 0.1736 |
|  | **1st** | **FODR1 vs FODR2** | 989 | 0.46866 | 0.41290 | 1.029x10-6 |  | * | 0.1640 |
|  | **mix2** | **FODR1 vs FODR2** | 989 | 0.3925 | 0.3529 | 3.889x10-5 |  | * | 0.09032 |
| **V2** | **mix3** | **FODR1 vs FODR2** | 989 | 0.3928 | 0.3154 | 7.791x10-18 |  | * | 0.2489 |
|  | **mix4** | **FODR1 vs FODR2** | 989 | 0.3834 | 0.2960 | 3.872x10-17 |  | * | 0.2447 |
|  | **mix5** | **FODR1 vs FODR2** | 989 | 0.3887 | 0.2844 | 3.227x10-26 |  | * | 0.3051 |
|  | **1st** | **FODR1 vs FODR2** | 1835 | 0.39725 | 0.38913 | 0.05439 |  |  | 0.02834 |
|  | **mix2** | **FODR1 vs FODR2** | 1835 | 0.3495 | 0.3400 | 0.2416 |  |  | 0.03382 |
| **IT** | **mix3** | **FODR1 vs FODR2** | 1835 | 0.3268 | 0.3117 | 0.001231 |  | * | 0.05913 |
|  | **mix4** | **FODR1 vs FODR2** | 1835 | 0.3186 | 0.2802 | 4.609x10-16 |  | * | 0.1654 |
|  | **mix5** | **FODR1 vs FODR2** | 1835 | 0.3025 | 0.3024 | 0.5245 |  |  | 0.01133 |
