# Supplementary material for: Neuronal Population Activity in Macaque Visual Cortices Dynamically Changes through Repeated Fixations in Active Free Viewing
Source: eNeuro. 2023 Oct 18;10(10):ENEURO.0086-23.2023. doi: 10.1523/ENEURO.0086-23.2023 (PMC10591287; doi:10.1523/ENEURO.0086-23.2023)
Supplement: Extended Data Table 6-1 — Comparison of ROC between saccade orders. The p-values were determined by the signed-rank test (two sided). The effect size is the Cliff’s δ effect size. Download Table 6-1, DOCX file. [file enu-eN-NWR-0086-23-s16.docx]

| **area** | **categories compared** | **n** | **mean1** | **mean2** | **p value**  **(signed-rank)** | **p < 0.05** | **p < 0.01** | **effect size** |
| --- | --- | --- | --- | --- | --- | --- | --- | --- |
| **V1 FODR1** | **1st vs mix2** | 5 | 0.75533 | 0.73361 | 0.06250 |  |  | 0.25 |
|  | **1st vs mix3** | 5 | 0.75533 | 0.75034 | 0.62500 |  |  | 0.125 |
|  | **1st vs mix4** | 5 | 0.75533 | 0.72937 | 0.06250 |  |  | 0.25 |
|  | **1st vs mix5** | 5 | 0.75533 | 0.71422 | 0.06250 |  |  | 0.25 |
| **V1 FODR2** | **1st vs mix2** | 5 | 0.76669 | 0.74328 | 0.06250 |  |  | 0.375 |
|  | **1st vs mix3** | 5 | 0.76669 | 0.72737 | 0.06250 |  |  | 0.625 |
|  | **1st vs mix4** | 5 | 0.76669 | 0.73604 | 0.06250 |  |  | 0.375 |
|  | **1st vs mix5** | 5 | 0.76669 | 0.73701 | 0.06250 |  |  | 0.625 |
| **V2 FODR1** | **1st vs mix2** | 10 | 0.7453 | 0.71316 | 0.01367 | * |  | 0.407 |
|  | **1st vs mix3** | 10 | 0.7453 | 0.70924 | 0.01953 | * |  | 0.432 |
|  | **1st vs mix4** | 10 | 0.7453 | 0.69944 | 1.953x10-3 |  | * | 0.556 |
|  | **1st vs mix5** | 10 | 0.7453 | 0.68518 | 3.906x10-3 |  | * | 0.601 |
| **V2 FODR2** | **1st vs mix2** | 10 | 0.74334 | 0.72178 | 0.083984 |  |  | 0.284 |
|  | **1st vs mix3** | 10 | 0.74334 | 0.6921 | 1.953x10-3 |  | * | 0.679 |
|  | **1st vs mix4** | 10 | 0.74334 | 0.70616 | 1.953x10-3 |  | * | 0.630 |
|  | **1st vs mix5** | 10 | 0.74334 | 0.67253 | 0.039062 | * |  | 0.556 |
| **IT FODR1** | **1st vs mix2** | 18 | 0.78764 | 0.7614 | 2.138x10-3 |  | * | 0.218 |
|  | **1st vs mix3** | 18 | 0.78764 | 0.75378 | 1.592x10-3 |  | * | 0.308 |
|  | **1st vs mix4** | 18 | 0.78764 | 0.74888 | 4.552x10-4 |  | * | 0.467 |
|  | **1st vs mix5** | 18 | 0.78764 | 0.7449 | 1.847x10-3 |  | * | 0.495 |
| **IT FODR2** | **1st vs mix2** | 18 | 0.78432 | 0.75673 | 5.357x10-4 |  | * | 0.301 |
|  | **1st vs mix3** | 18 | 0.78432 | 0.74852 | 1.009x10-3 |  | * | 0.481 |
|  | **1st vs mix4** | 18 | 0.78432 | 0.74085 | 2.332x10-4 |  | * | 0.529 |
|  | **1st vs mix5** | 18 | 0.78432 | 0.74091 | 3.2701x10-4 |  | * | 0.522 |
