# Supplementary material for: Neuronal Population Activity in Macaque Visual Cortices Dynamically Changes through Repeated Fixations in Active Free Viewing
Source: eNeuro. 2023 Oct 18;10(10):ENEURO.0086-23.2023. doi: 10.1523/ENEURO.0086-23.2023 (PMC10591287; doi:10.1523/ENEURO.0086-23.2023)
Supplement: Extended Data Table 6-2 — Comparison of ROC between saccade orders. The p-values were determined by the signed-rank test (two sided). The effect size is the Cliff’s δ effect size. Download Table 6-2, DOCX file. [file enu-eN-NWR-0086-23-s17.docx]

| **area** | **fixations** | **category to compare** | **n** | **mean1** | **mean2** | **p value**  **(signed-rank)** | **p<0.05** | **p<0.01** | **effect size** |
| --- | --- | --- | --- | --- | --- | --- | --- | --- | --- |
| **V1** | **mix2** | **FODR1 vs FODR2** | 5 | 0.75533 | 0.76669 | 0.625 |  |  | 0.25 |
|  | **mix3** | **FODR1 vs FODR2** | 5 | 0.73361 | 0.74328 | 0.625 |  |  | 0.125 |
|  | **mix4** | **FODR1 vs FODR2** | 5 | 0.75034 | 0.72737 | 0.3125 |  |  | 0.25 |
|  | **mix5** | **FODR1 vs FODR2** | 5 | 0.72937 | 0.73604 | 1 |  |  | 0.25 |
| **V2** | **mix2** | **FODR1 vs FODR2** | 10 | 0.74530 | 0.74334 | 0.84570 |  |  | 0.40741 |
|  | **mix3** | **FODR1 vs FODR2** | 10 | 0.71316 | 0.72178 | 0.625 |  |  | 0.43210 |
|  | **mix4** | **FODR1 vs FODR2** | 10 | 0.70929 | 0.69210 | 0.32227 |  |  | 0.55556 |
|  | **mix5** | **FODR1 vs FODR2** | 10 | 0.69944 | 0.70616 | 0.43164 |  |  | 0.60494 |
| **IT** | **mix2** | **FODR1 vs FODR2** | 18 | 0.78764 | 0.78432 | 0.84463 |  |  | 0.21710 |
|  | **mix3** | **FODR1 vs FODR2** | 18 | 0.76140 | 0.75673 | 0.49966 |  |  | 0.30710 |
|  | **mix4** | **FODR1 vs FODR2** | 18 | 0.75378 | 0.74852 | 0.34911 |  |  | 0.46713 |
|  | **mix5** | **FODR1 vs FODR2** | 18 | 0.74888 | 0.74085 | 0.32714 |  |  | 0.49481 |
