# Supplementary material for: Neuronal Population Activity in Macaque Visual Cortices Dynamically Changes through Repeated Fixations in Active Free Viewing
Source: eNeuro. 2023 Oct 18;10(10):ENEURO.0086-23.2023. doi: 10.1523/ENEURO.0086-23.2023 (PMC10591287; doi:10.1523/ENEURO.0086-23.2023)
Supplement: Extended Data Table 7-1 — Comparison of discrimination accuracy by LDA between first and later fixations. The p-values were determined by the signed-rank test (two sided). The effect size is the Cliff’s δ effect size. Download Table 7-1, DOCX file. [file enu-eN-NWR-0086-23-s18.docx]

| **area and period** | **categories compared** | **n** | **mean1** | **mean2** | **p value**  **(signed-rank)** | **p < 0.05** | **p < 0.01** | **effect size** |
| --- | --- | --- | --- | --- | --- | --- | --- | --- |
| **V1/V2 FODR1** | **1st vs mix2-3** | 11 | 0.02311 | 0.006710 | 0.1748 |  |  | 0.3884 |
|  | **1st vs mix4-6** | 11 | 0.02311 | 0.01998 | 0.6377 |  |  | 0.008265 |
| **V1/V2 FODR2** | **1st vs mix2-3** | 11 | 0.03064 | 0.01178 | 0.08301 |  |  | 0.4380 |
|  | **1st vs mix4-6** | 11 | 0.03064 | 0.02551 | 0.4648 |  |  | 0.1074 |
| **IT FODR1** | **1st vs mix2-3** | 18 | 0.04696 | 0.02910 | 0.03467 | * |  | 0.2593 |
|  | **1st vs mix4-6** | 18 | 0.04696 | 0.02719 | 0.006491 |  | * | 0.3210 |
| **IT FODR2** | **1st vs mix2-3** | 18 | 0.03014 | 0.02201 | 0.2668 |  |  | 0.05556 |
|  | **1st vs mix4-6** | 18 | 0.03014 | 0.02594 | 0.9826 |  |  | 0.1111 |
