# Supplementary material for: Neuronal Population Activity in Macaque Visual Cortices Dynamically Changes through Repeated Fixations in Active Free Viewing
Source: eNeuro. 2023 Oct 18;10(10):ENEURO.0086-23.2023. doi: 10.1523/ENEURO.0086-23.2023 (PMC10591287; doi:10.1523/ENEURO.0086-23.2023)
Supplement: Extended Data Table 7-2 — Comparison of discrimination accuracy by LDA between FODR1 and FODR2. The p-values were determined by the signed-rank test (two sided). The effect size is the Cliff’s δ effect size. Download Table 7-2, DOCX file. [file enu-eN-NWR-0086-23-s19.docx]

| **area and period** | **fixation** | **categories compared** | **n** | **mean1** | **mean2** | **p value**  **(signed-rank)** | **p < 0.05** | **p < 0.01** | **effect size** |
| --- | --- | --- | --- | --- | --- | --- | --- | --- | --- |
|  | **1st** | **FODR1 vs FODR2** | 11 | 0.02311 | 0.03064 | 0.3652 |  |  | 0.2562 |
| V1/V2 | **mix2-3** | **FODR1 vs FODR2** | 11 | 0.00671 | 0.0117 | 0.2783 |  |  | 0.1240 |
|  | **mix 4-6** | **FODR1 vs FODR2** | 11 | 0.01998 | 0.02551 | 0.5195 |  |  | 0.1240 |
|  | **1st** | **FODR1 vs FODR2** | 18 | 0.04696 | 0.03014 | 0.0050 |  | * | 0.3210 |
| IT | **mix2-3** | **FODR1 vs FODR2** | 18 | 0.0291 | 0.02201 | 0.2145 |  |  | 0.1543 |
|  | **mix 4-6** | **FODR1 vs FODR2** | 18 | 0.02719 | 0.02594 | 0.8107 |  |  | 0.0185 |
